# Biosynthesis of the Inner Core of *Bordetella pertussis* Lipopolysaccharides: Effect of Mutations on LPS Structure, Cell Division, and Toll-like Receptor 4 Activation

**DOI:** 10.3390/ijms242417313

**Published:** 2023-12-09

**Authors:** Jesús Pérez-Ortega, Ria van Boxtel, Michel Plisnier, Dominique Ingels, Nathalie Devos, Steven Sijmons, Jan Tommassen

**Affiliations:** 1 Section Molecular Microbiology, Department of Biology, Faculty of Science, Utrecht University, 3584 CH Utrecht, The Netherlands; j.perezortega@uu.nl (J.P.-O.); h.a.m.tommassen@uu.nl (R.v.B.); 2 Institute of Biomembranes, Utrecht University, 3584 CH Utrecht, The Netherlands; 3 Vaccines Research & Development, GSK, 1330 Rixensart, Belgium; michel.plisnier@gsk.com (M.P.); dominique.c.ingels@gsk.com (D.I.); nathalie.i.devos@gsk.com (N.D.); steven.x.sijmons@gsk.com (S.S.)

**Keywords:** *Bordetella pertussis*, cell division, 3-deoxy-D-*manno*-oct-2-ulosonic acid (Kdo), endotoxin, Kdo kinase, Kdo transferase, lipid A phosphatase, lipopolysaccharide, phosphoethanolamine transferase, Toll-like receptor 4

## Abstract

Previously developed whole-cell vaccines against *Bordetella pertussis*, the causative agent of whooping cough, appeared to be too reactogenic due to their endotoxin content. Reduction in endotoxicity can generally be achieved through structural modifications in the lipid A moiety of lipopolysaccharides (LPS). In this study, we found that dephosphorylation of lipid A in *B. pertussis* through the heterologous production of the phosphatase LpxE from *Francisella novicida* did, unexpectedly, not affect Toll-like receptor 4 (TLR4)-stimulating activity. We then focused on the inner core of LPS, whose synthesis has so far not been studied in *B. pertussis*. The *kdtA* and *kdkA* genes, responsible for the incorporation of a single 3-deoxy-D-*manno*-oct-2-ulosonic acid (Kdo) residue in the inner core and its phosphorylation, respectively, appeared to be essential. However, the Kdo-bound phosphate could be replaced by a second Kdo after the heterologous production of *Escherichia coli kdtA*. This structural change in the inner core affected outer-core and lipid A structures and also bacterial physiology, as reflected in cell filamentation and a switch in virulence phase. Furthermore, the *eptB* gene responsible for the non-stoichiometric substitution of Kdo-bound phosphate with phosphoethanolamine was identified and inactivated. Interestingly, the constructed inner-core modifications affected TLR4-stimulating activity. Whereas endotoxicity studies generally focus on the lipid A moiety, our data demonstrate that structural changes in the inner core can also affect TLR4-stimulating activity.

## 1. Introduction

*Bordetella pertussis* is a strictly human pathogen responsible for the respiratory infection known as whooping cough or pertussis. Introduction of whole-cell pertussis vaccines (wP) around the 1940s led to a drastic reduction in pertussis cases. However, in the 1990s, these vaccines were replaced by acellular formulations (aP) because of safety concerns caused by the reactogenicity of wP [1]. In countries where aP vaccination was introduced, the number of pertussis cases is on the rise. This can be at least partially attributed to the rapid waning of the immune protection evoked by these vaccines, as well as by their failure to prevent mucosal colonization and transmission [2]. Thus, there is a need for new vaccines, for which the development of novel wP with reduced reactogenicity could be an option.

**Figure 1:**
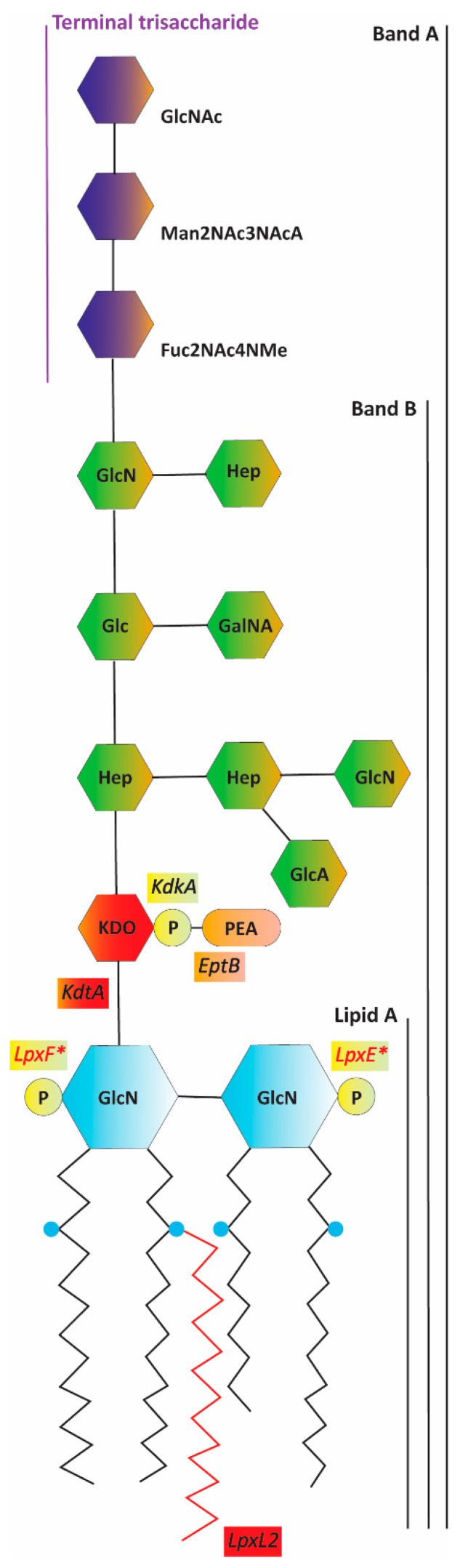
Schematic representation of *B. pertussis* LPS [19]. The various sugar units are depicted with hexagons; the phosphate groups, with yellow circles; the PEA substitution, with a rounded rectangle; and the hydroxylation of fatty acids, with blue circles. Also, the enzymes that are discussed in this paper are indicated next to and labeled with the same color as the groups they add (enzymes in black characters) or remove (enzymes in red characters and labeled with an asterisk). The secondary C_14_ chain at position 2′, which is inserted by the acylase LpxL2, is indicated in red. The portions of LPS corresponding to lipid A, band A, band B, and the terminal trisaccharide are indicated with vertical lines. Abbreviations: GlcN, glucosamine; P, phosphate; Kdo, 3-deoxy-D-*manno*-oct-2-ulosonic acid; PEA, phosphoethanolamine; Hep, heptose; GlcA, glucuronic acid; Glc, glucose; GalNA, galactosaminuronic acid; Fuc2NAc4NMe, β-L-2-acetamido-4-methylamino-fucose; Man2NAc3NAcA, β-2-acetamido-3-acetamido-2,3-dideoxy-mannuronic acid; GlcNAc, α-*N*-acetyl-GlcN.

The main culprits of the reactogenicity of wP are lipopolysaccharides (LPS, also known as endotoxins). LPS are the major components of the outer leaflet of the outer membrane (OM) of Gram-negative bacteria [3]. They consist of three domains, i.e., lipid A, a core oligosaccharide, and a polysaccharide known as O-antigen [4]. The latter is absent in the LPS of *B. pertussis* and of some other species, and the LPS of these bacteria are also referred to as lipooligosaccharides (LOS). Innate immune cells recognize LPS during infection by Gram-negative bacteria and release pro-inflammatory cytokines, such as TNFα and IL-1β, which activate immune defenses. However, overstimulation of the immune system can cause serious damage with sometimes fatal consequences [5]. This endotoxic response is also responsible for the reactogenicity of whole-cell vaccines against Gram-negative bacteria. LPS, mainly their lipid A moiety, are recognized by a heterodimeric receptor composed of Toll-like receptor 4 (TLR4) and myeloid differentiation factor 2 (MD-2) [4]. The binding of LPS induces the dimerization of the receptor, with LPS being the bridge between the two heterodimers in the resulting tetramer [6]. Lipid A is a glucosamine (GlcN) disaccharide substituted with phosphates at positions 1 and 4′ and acylated at positions 2, 2′, 3, and 3′ with β-hydroxylated fatty acids, which can be substituted with secondary acyl chains (Figure 1) [4]. Structural modification of lipid A can affect the activation of TLR4 and, thereby, of cytokine production. Usually, reduction in the numbers of fatty acids or phosphates results in reduced endotoxicity [7,8].

In recent decades, genetic engineering has been applied in *B. pertussis* for the development of novel wP vaccines with reduced endotoxicity [9,10]. These studies have focused on the number and the length of the acyl chains of LPS. *B. pertussis* lipid A presents two phosphate groups (Figure 1), which can be substituted with GlcN residues. The glycosyltransferase responsible for the introduction of this sugar is LgmB. In *B. pertussis*, *lgmB* expression seems to vary among strains, but its activity is usually low or even absent [11]. To our knowledge, no genetic engineering efforts to reduce endotoxicity in *B. pertussis* have ever targeted the phosphates in lipid A. Some bacterial species, including *Francisella novicida*, produce the phosphatases LpxE and/or LpxF, which remove the phosphates at positions 1 and 4′, respectively, of lipid A (Figure 1). These phosphatases are integral inner-membrane proteins and present their active site towards the periplasm. Since phosphates contribute to receptor binding and dimerization by forming ionic interactions with clusters of positively charged residues in TLR4 and MD-2 [6], the activity of phosphatases generates LPS forms with reduced endotoxicity [7]. Upon expression in *Escherichia coli* and *Salmonella enterica* serovar Typhimurium (*S*. Typhimurium), the LpxE of *F. novicida* (LpxE_Fn_) has been shown to effectively remove the phosphate at position 1 of heterologous lipid A [12]. However, *F. novicida* LpxF (LpxF_Fn_) selectively dephosphorylates tetra- and penta-acylated lipid A molecules and thus has no effect on wild-type lipid A from *E. coli*, which is hexa-acylated [13]. Heterologous expression of *lpxE_Fn_* has proved to efficiently reduce endotoxicity in, for example, *S*. Typhimurium [14]. Such strategies are currently used in vaccine development to reduce the endotoxicity of bacterial cells [15,16], but they have not been applied for *B. pertussis* so far.

Aside from the phosphates in lipid A, negatively charged residues in the inner core seem to facilitate TLR4 signaling in at least some bacterial species [17]. In *B. pertussis* LPS, a phosphate group is present at position 4 of the single 3-deoxy-D-*manno*-oct-2-ulosonic acid (Kdo) present in the inner-core region (Figure 1) [18]. In turn, this phosphate is non-stoichiometrically substituted with phosphoethanolamine (PEA) [19] (Figure 1). The kinase responsible for the insertion of the phosphate group in Kdo and the specific PEA transferase are generically called KdkA and EptB, respectively [20,21] (Figure 1). However, these enzymes have not been characterized in *B. pertussis* so far, and the *eptB* gene has not even been identified. As mentioned above, *B. pertussis* LPS present a single Kdo (KdoI), which is transferred by the monofunctional Kdo transferase KdtA (also known as WaaA) [22]. In contrast, the KdtA of *E. coli* (KdtA_Ec_) and of many other Gram-negative bacteria introduces two Kdo residues [4,23]. The location of the second Kdo (KdoII) is position 4 of KdoI, i.e., the same position where the phosphate substitution takes place in *B. pertussis* KdoI.

The outer core of *B. pertussis* LPS presents a terminal non-repeating trisaccharide (Figure 1) consisting of GlcNAc, β-2,3-diacetamido-2,3-dideoxy-mannuronic acid (Man2NAc3NAcA), and β-2-acetamido-4-N-methyl-2,4-dideoxy-fucose (Fuc2NAc4NMe) [19], all synthesized and attached by enzymes encoded in the *wlb* operon [24,25]. This trisaccharide is synthesized separately and is added en bloc to the core. It is responsible for the detection of two bands when *B. pertussis* LPS are analyzed using SDS-PAGE. Band A corresponds to the entire LPS structure, i.e., lipid A and a branched dodecasaccharide core, while band B lacks the terminal trisaccharide (Figure 1).

In this study, we aimed at dephosphorylating *B. pertussis* lipid A through the heterologous production of the phosphatases LpxE_Fn_ and LpxF_Fn_. In addition, we modified the environment of the core Kdo sugar through the heterologous production of the bi-functional KdtA of *E. coli* and through the inactivation of *eptB*, and the effects of the genetic modifications on LPS structure, bacterial physiology, and TLR4 activation were determined.

## 2. Results

### 2.1. Targeting the Phosphate Groups of B. pertussis Lipid A

**Figure 2:**
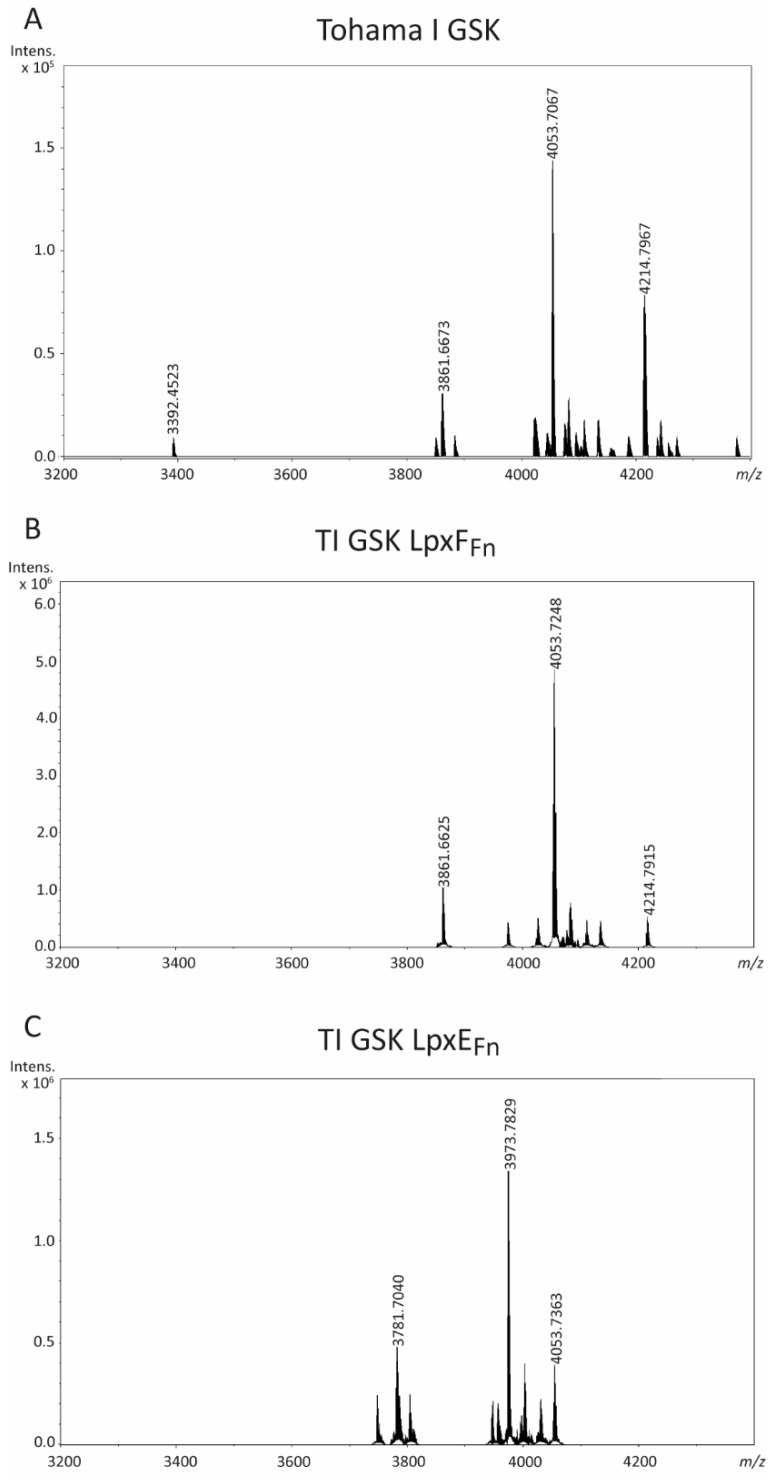
LC-MS analysis of LPS of *B. pertussis* strain Tohama I GSK (**A**), and its LpxF_Fn_-producing (**B**) and LpxE_Fn_-producing (**C**) derivatives. The measured monoisotopic mass of the peaks characterized is indicated. The peak at *m*/*z* 4053.7 corresponds to the expected structure of wild-type LPS. For interpretation of the other peaks, see Table 1.

The removal of the phosphate groups of lipid A was tackled through the heterologous production of the phosphatases LpxE_Fn_ and LpxF_Fn_ in the *B. pertussis* strain Tohama I GSK. Codon-optimized versions of the corresponding genes under the control of the *lac* promoter were introduced into this strain on plasmid pMMB67EH. To evaluate the alterations in LPS composition, LPS were analyzed using LC-MS in negative-ion mode. This analysis revealed three major LPS species in the parental strain (Figure 2A; see structural details in Table 1). The predominant peak at *m*/*z* 4053.7 was attributed to the characteristic penta-acylated and bis-phosphorylated lipid A species that is typically found in *B. pertussis* [26] substituted with a complete core structure and containing a PEA substitution on the Kdo-bound phosphate (Figure 1). The peak at *m*/*z* 3861.7 corresponds to the same structure but with the loss of a heptose, indicating that the heptose in the outer-core region (Figure 1) is a non-stoichiometric substitution. The third peak at *m*/*z* 4214.8 represents the major peak, with a GlcN substitution on one of the phosphate groups of lipid A. A minor peak at *m*/*z* 3392.5 represents the predominant peak (*m*/*z* 4053.7) but lacks the terminal trisaccharide.

The LPS of the strain producing LpxF_Fn_, designated as TI GSK LpxF_Fn_, showed no relevant modifications compared with the wild-type LPS, except that the peak representing the GlcN modification is substantially smaller (Figure 2B). Apparently, this phosphatase is not active on *B. pertussis* LPS as a substrate. In contrast, the LPS of the strain producing LpxE_Fn_ showed partial dephosphorylation, and this strain was further improved by inserting the *lpxE*_Fn_ gene under the control of the strong, constitutive *B. pertussis ompP* promoter into the chromosome in place of the *lgmB* locus. As the GlcN substitution on the 1-phosphate presumably hinders the phosphatase activity of LpxE_Fn_, the inactivation of *lgmB* was expected to enhance dephosphorylation. The LC-MS analysis of the LPS of the strain, designated as TI GSK LpxE_Fn_, showed a major peak at *m*/*z* 3973.8, which corresponds to the molecular ion at *m*/*z* 4053.7 of wild-type LPS with the expected loss of a phosphate (Figure 2C). Similarly, the new peak at *m*/*z* 3781.7 represents the LPS missing one heptose (*m*/*z* 3861.7 in wild-type LPS) with the additional loss of a phosphate. A peak reflecting the additional GlcN substitution was not observed as expected because of the inactivation of the *lgmB* gene. Only a small portion of wild-type LPS (*m*/*z* 4053.7) could be detected (Figure 2C).

To determine whether the dephosphorylation of the LPS structure reduces the TLR4-stimulating activity of *B. pertussis* whole cells, TLR4 activation was tested in HEK-Blue reporter cells expressing human TLR4. Unexpectedly, heat-killed whole cells of TI GSK LpxE_Fn_ and of its parental strain stimulated TLR4 to similar levels (Figure S1), despite the efficient LPS dephosphorylation observed in the LC-MS analysis.

### 2.2. Modification of the Inner Core of B. pertussis LPS Using KdtA_Ec_

**Figure 3:**
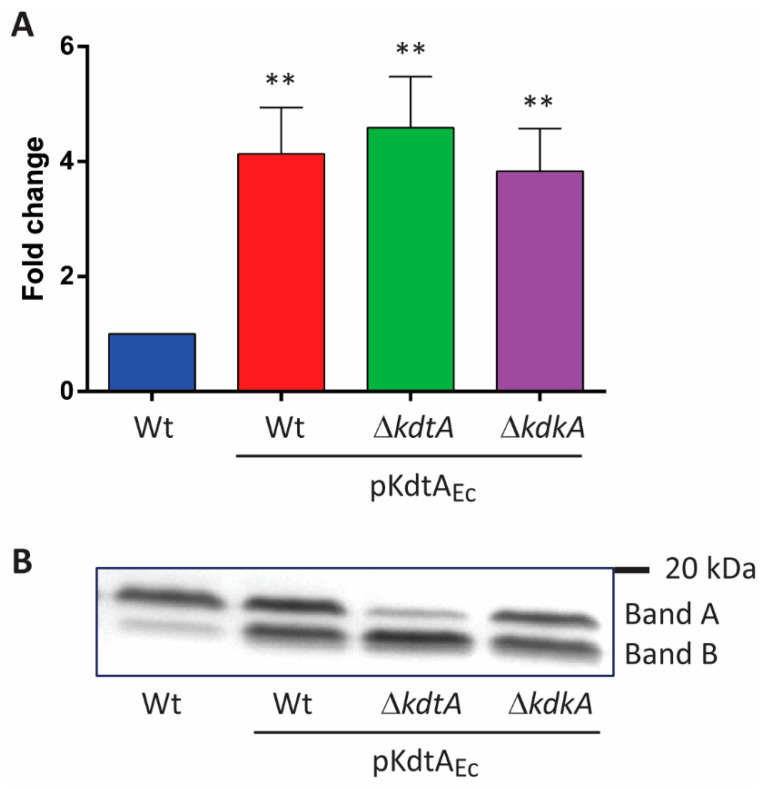
KdtA_Ec_ expression affects the structure of *B. pertussis* LPS. (**A**) Kdo content was quantified in OMs isolated from strain B213 and its pKdtA_Ec_-containing derivatives. Fold changes relative to the wild type (Wt) were calculated from the Kdo concentrations obtained. Data represent means and standard deviations of three independent experiments performed in duplicate. Statistically significant differences from the wild type are indicated with asterisks (** *p* < 0.01). (**B**) LPS from one of the OM samples of each strain were analyzed using SDS-PAGE and visualized using silver staining. The positions of band A, band B, and a molecular-weight marker protein are indicated on the right.

As the removal of the 1-phosphate did not affect TLR4 activation, we continued by investigating the inner-core region. Previously, it has been suggested that the negative charges in the inner core of the LPS of *Capnocytophaga canimorsus* are important for TLR4 activation [17]. The LPS of this species present a single Kdo and lack one of the phosphates of lipid A, while the other phosphate is replaced by PEA. Similarly, the inner core of *B. pertussis* LPS can affect TLR4 signaling. Therefore, we targeted this region of LPS. The inactivation of the *kdtA* (locus tag BP0095) and *kdkA* (BP2349) genes, which encode the Kdo transferase and Kdo kinase, respectively, failed, indicating that these genes are essential for the viability of the cells. Consequently, we first tried to change the inner core by introducing KdoII in *B. pertussis* LPS. To this end, plasmid pKdtA_Ec_ containing the gene for bi-functional KdtA from *E. coli* was constructed and introduced into strain B213, another Tohama I derivative, through conjugation. Kdo assays on OM preparations of the resulting transconjugants showed a significant increase in Kdo content (Figure 3A). We considered the possibility that the mono-functional KdtA of *B. pertussis*, expressed by the chromosome, or the physical hindrance resulting from the phosphate at position 4 of KdoI, i.e., where KdoII is expected to be inserted, could reduce the efficiency of KdoII insertion. Therefore, we tried to delete *kdtA* and *kdkA* in strain B213 carrying pKdtA_Ec_. Whilst these genes could not be knocked out in strain B213, they could be knocked out in the strain carrying the plasmid. However, Kdo assays did not indicate a further increase in the incorporation of KdoII (Figure 3A).

The analysis of LPS using SDS-PAGE showed structural modifications, reflected in altered electrophoretic mobility, in all the strains producing KdtA_Ec_. Whilst the vast majority of LPS in the wild-type strain migrated in the position of band A, large amounts of LPS produced in the recombinant strains migrated faster and appeared at the level of band B (Figure 3B). The KdtA_Ec_-producing *kdtA* mutant showed the most extensive switch to the form with higher electrophoretic mobility. The faster migration of the new LPS form suggests a reduction in the size of LPS despite the KdoII insertion.

**Figure 4:**
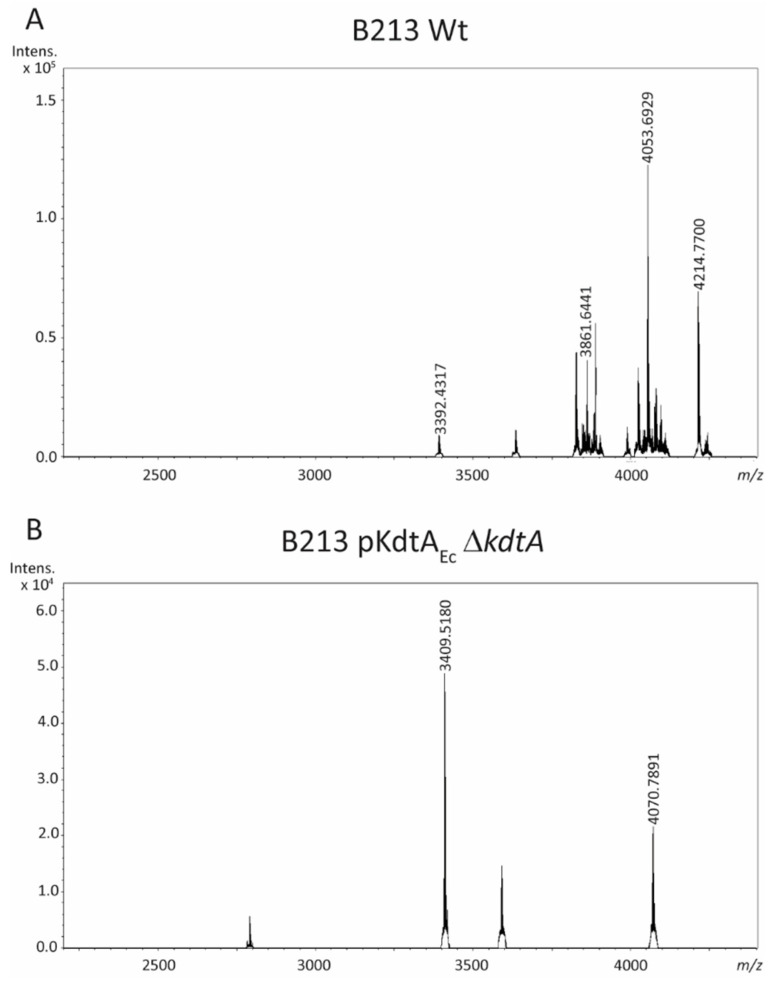
LC-MS analysis of LPS of *B. pertussis* strain B213 (**A**) and its Δ*kdtA*-derivative containing pKdtA_Ec_ (**B**). The measured monoisotopic mass of the peaks characterized is indicated. The peak at *m*/*z* 4053.7 corresponds to the wild-type LPS structure. For interpretation of the other peaks, see Table 1.

The LC-MS analysis of the LPS of strain B213 (Figure 4A) presented the same four peaks as described above for strain Tohama I GSK (Figure 2A; compare in Table 1). The *kdtA* mutant derivative of B213 pKdtA_Ec_ showed a major peak at *m*/*z* 3409.5, corresponding to the molecular ion at *m*/*z* 4053.7 of the parental strain (wild-type structure), but containing the additional KdoII and lacking the phosphate-PEA (PPEA) substitution and the terminal trisaccharide (Figure 4B and Table 1). A smaller peak at *m*/*z* 4070.8 corresponds to the major peak at *m*/*z* 3409.5 but includes the terminal trisaccharide.

After evidencing the possibility of inserting KdoII in *B. pertussis* LPS, we proceeded by combining this modification with LpxE_Fn_ production. First, pKdtA_Ec_ was introduced in strain TI GSK LpxE_Fn_, and subsequently, *kdtA* and *kdkA* mutants were generated. As before (Figure 3B), the production of KdtA_Ec_ in TI GSK LpxE_Fn_ resulted in the modification of the LPS structure, as evidenced by SDS-PAGE results, and the chromosomal *kdtA* and *kdkA* mutations strongly enhanced the relatively mild effect observed after the introduction of pKdtA_Ec_ (Figure S2).

**Figure 5:**
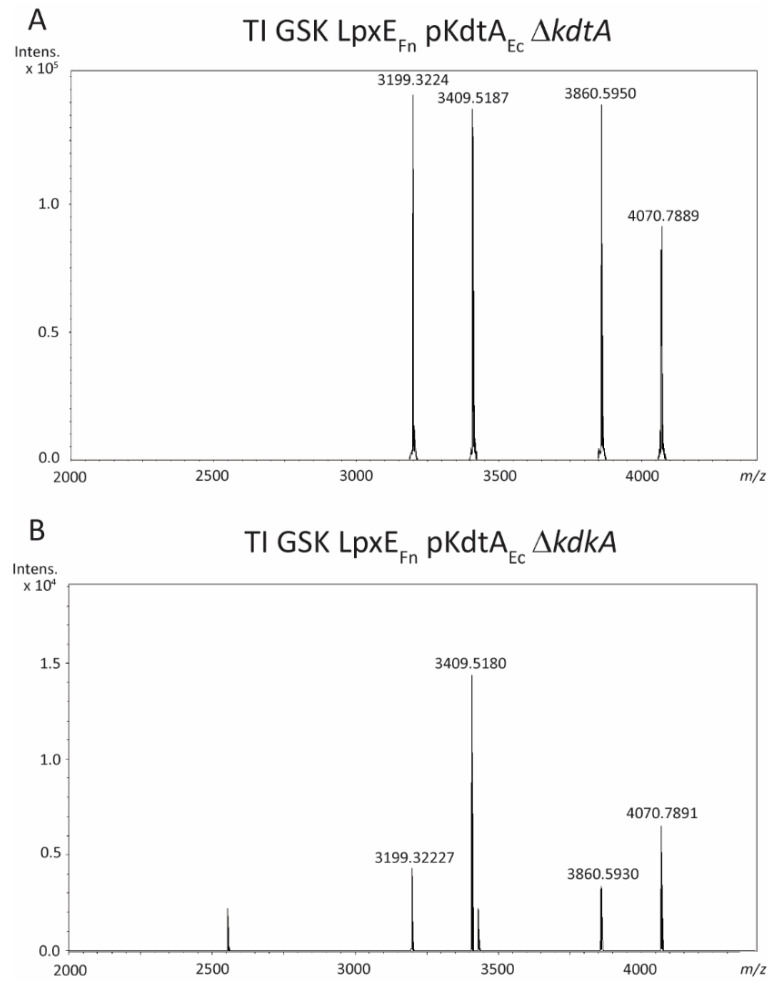
LC-MS analysis of LPS of strains TI GSK LpxE_Fn_ pKdtA_Ec_ Δ*kdtA* (**A**) and TI GSK LpxE_Fn_ pKdtA_Ec_ Δ*kdkA* (**B**). Measured monoisotopic mass of the peaks characterized is indicated. The major peaks at *m*/*z* 3409.5 and *m*/*z* 3199.3 correspond to the LPS form with the additional KdoII but lacking the PPEA substitution and the final trisaccharide, and the same LPS form with the additional loss of a C_14_ acyl chain, respectively. Peaks corresponding to dephosphorylated forms were not detected.

The LC-MS analysis of LPS from the *kdtA* mutant of strain TI GSK LpxE_Fn_ containing pKdtA_Ec_ (Figure 5A) showed prominent peaks at *m*/*z* 3409.5 and 4070.8, which correspond to those detected in the *kdtA* mutant of strain B213 pKdtA_Ec_ (Figure 4B; compare in Table 1). Apparently, the introduction of KdoII prevented the dephosphorylation of lipid A by LpxE_Fn_. Two additional peaks at *m*/*z* 3199.4 and 3860.6 (Figure 5A) represent the same structures as *m*/*z* 3409.5 and 4070.8, respectively, but with the additional loss of a C_14_ acyl chain (Table 1). Similarly, the *kdkA* mutant of strain TI GSK LpxE_Fn_ containing pKdtA_Ec_ showed the same peaks as identified in the *kdtA* mutant, although the peaks representing the deacylated forms were less abundant in this case (Figure 5B).

### 2.3. Phenotypic Characterization of KdtA_Ec_-Producing Strains

**Figure 6:**
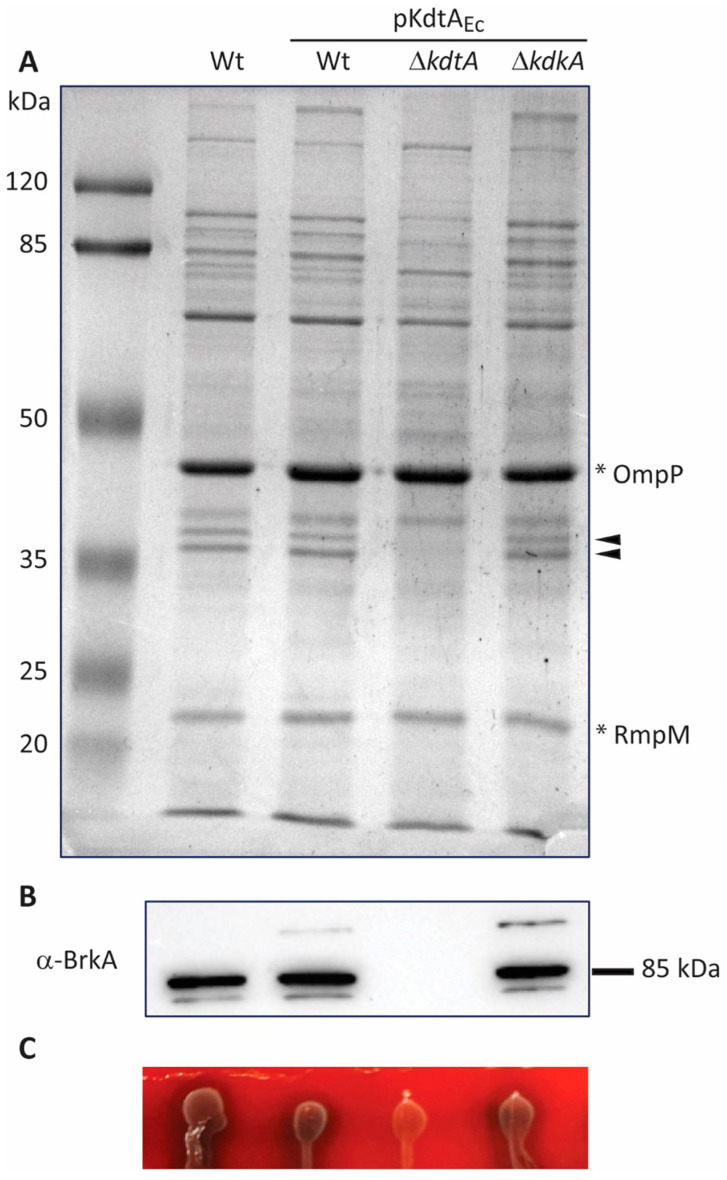
Comparison of protein content of preparations from *B. pertussis* strain B213 (Wt) and its pKdtA_Ec_-containing derivatives. (**A**) Isolated OMs were analyzed using SDS-PAGE. The major OM protein, porin OmpP, and the porin-associated periplasmic protein RmpM are indicated on the right with asterisks. Arrowheads indicate two protein bands that are lost in the pKdtA_Ec_-containing *kdtA* mutant. Molecular-weight markers are shown on the left. (**B**) OM proteins were separated using SDS-PAGE, and Western blot analysis was performed with antibodies directed against the autotransporter BrkA. The position of a molecular-weight marker protein is indicated on the right. Only the relevant part of the blot is shown. (**C**) Hemolytic capacity of the strains. Drops from bacterial cultures were plated on BG-blood agar and incubated for 48 h. The dark halo indicates hemolytic activity.

Compared with their parental strain, all strains producing KdtA_Ec_ showed reduced growth kinetics in liquid medium (Figure S3). The OM protein profiles of strain B213 and its derivatives were very similar except for the pKdtA_Ec_-containing *kdtA* mutant (Figure 6A). In the latter, two prominent bands of around 36–38 kDa (indicated by arrowheads in Figure 6A) were lost. These bands have been shown to correspond to the β-barrel domains of autotransporters such as BrkA, Prn, and Tcf [27,28]. Notable differences were also observed in the banding pattern around 80–115 kDa (Figure 6A); these bands are probably related to the passenger domains of autotransporters. The production of specific OM proteins was further analyzed with Western blotting. The autotransporter BrkA could be detected in strain B213 and its derivatives, except in the pKdtA_Ec_-containing *kdtA* mutant (Figure 6B). In contrast, other OM proteins, such as the major porin OmpP and the porin-associated periplasmic protein RmpM, also known as OmpA [29], appeared to be present in similar amounts in all strains (Figure S4).

As *brkA* expression is dependent on the two-component regulatory system Bvg [28], we considered the possibility of a phase switch from Bvg^+^ to Bvg^−^ in the pKdtA_Ec_-containing *kdtA* mutant strain. To investigate this possibility, we studied another Bvg-dependent property, i.e., hemolysis [30]. While the wild-type strain showed hemolysis on BG-blood plates, which indicates that it was growing in the Bvg^+^ phase, the *kdtA* mutant did not show this phenotype (Figure 6C), suggesting a switch to the Bvg^−^ phase. The other two pKdtA_Ec_-containing strains showed hemolysis similar to that of their parental strain. In the genetic background of the LpxE_Fn_-expressing derivative of strain Tohama I GSK containing pKdtA_Ec_, the production of BrkA (Figure S5A) and hemolysis on BG-blood plates (Figure S5B) were lost in both the *kdtA* and the *kdkA* mutant.

**Figure 7:**
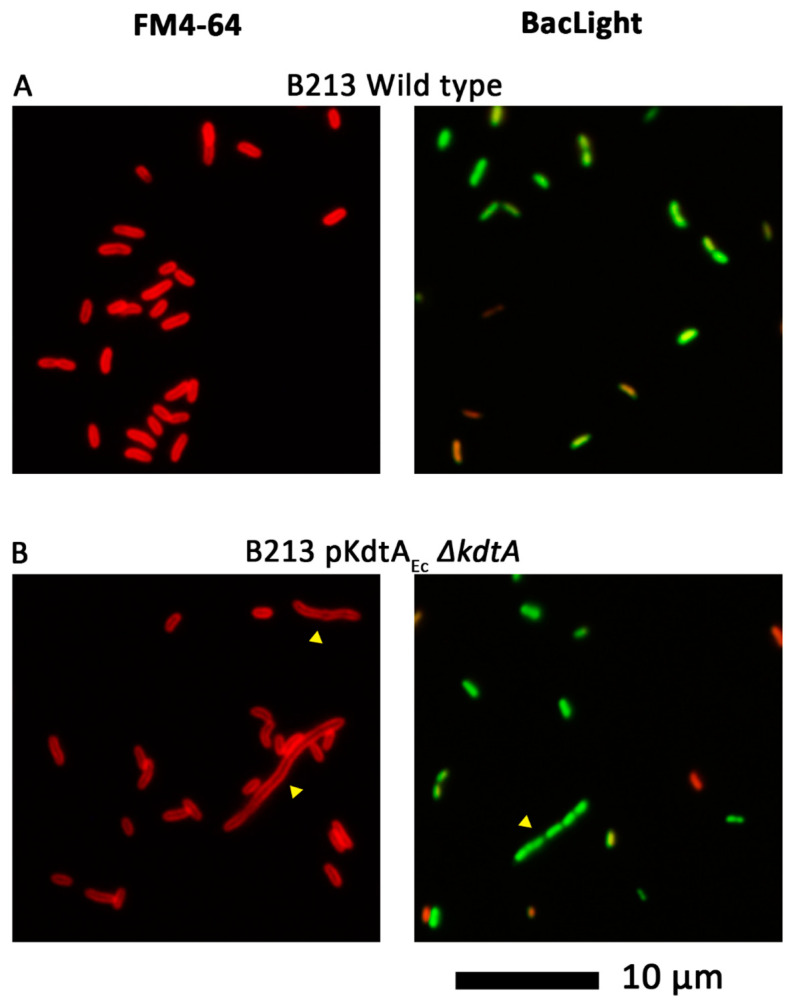
KdtA_Ec_ production affects cell division. Bacterial cultures of strain B213 (**A**) and its pKdtA_Ec_ Δ*kdtA* derivative (**B**) were stained with either the fluorescent dye FM4-64 (**left panels**) or Dead/Live BacLight kit (**right panels**) and studied using fluorescence microscopy. Scale bar represents 10 µm. Examples of cells showing elongated cell chains are indicated with yellow arrowheads.

The reduced growth of the pKdtA_Ec_-containing strains could be due to impaired cell division. To investigate this possibility, cell morphology was studied using fluorescence microscopy after staining bacteria with either the fluorescent membrane dye FM4-64 or the Live/Dead BacLight kit, which contains two different nucleic acid stains. As expected, wild-type strain B213 displayed short rods (Figure 7A). Upon production of KdtA_Ec_ and inactivation of the chromosomal copy of *kdtA*, elongated cells were detected (indicated with yellow arrowheads in Figure 7B). As the nucleoids appeared to be separated in these filaments (Figure 7B, right panel) but the membrane dye did not indicate the presence of septa in such elongated cells (Figure 7B, left panel), these results indicate a defect early in cell division, probably at the level of the invagination of the cell membranes.

Cells producing LpxE_Fn_ appeared shorter and more rounded than the classic wild-type structure of Tohama I, and occasionally, short chains of cells were observed (purple and blue arrowheads, respectively, in Figure S6A). Cells producing both LpxE_Fn_ and KdtA_Ec_ were elongated and formed chains (yellow arrowheads in Figure S6B), like the phenotype observed when only KdtA_Ec_ was produced (Figure 7B), which was to be expected, as LpxE_Fn_-mediated lipid A dephosphorylation does not take place when KdoII is introduced (Figure 5). However, invaginations of the membranes could generally be observed in these cells (Figure S6B, left panel). The nucleoids also showed irregularities at the edge, suggesting defective compaction (white arrowheads in Figure S6B, right panel), which was not observed in the parental strain (Figure S6A, right panel).

### 2.4. Identification and Characterization of the eptB Gene in B. pertussis

Our MS analysis showed that the phosphate group attached to Kdo of LPS is effectively substituted with PEA (Figure 2A and Figure 4A). However, a putative *eptB* gene encoding the PEA transferase has not yet been described in *B. pertussis*. We selected two possible candidates for *eptB*, i.e., BP2327 and BP3136. BP2327 is located in a cluster of genes involved in core biosynthesis, next to BP2328-BP2331 [31], and the encoded protein shows some homology to lipid A PEA transferases (EptA). However, PEA substitutions at the phosphate groups of *B. pertussis* lipid A have never been described. Like the BP2327 product, the BP3136 protein was classified as a protein with an EptB-like structure including a sulfatase domain; thus, it may have a similar function [32,33].

**Figure 8:**
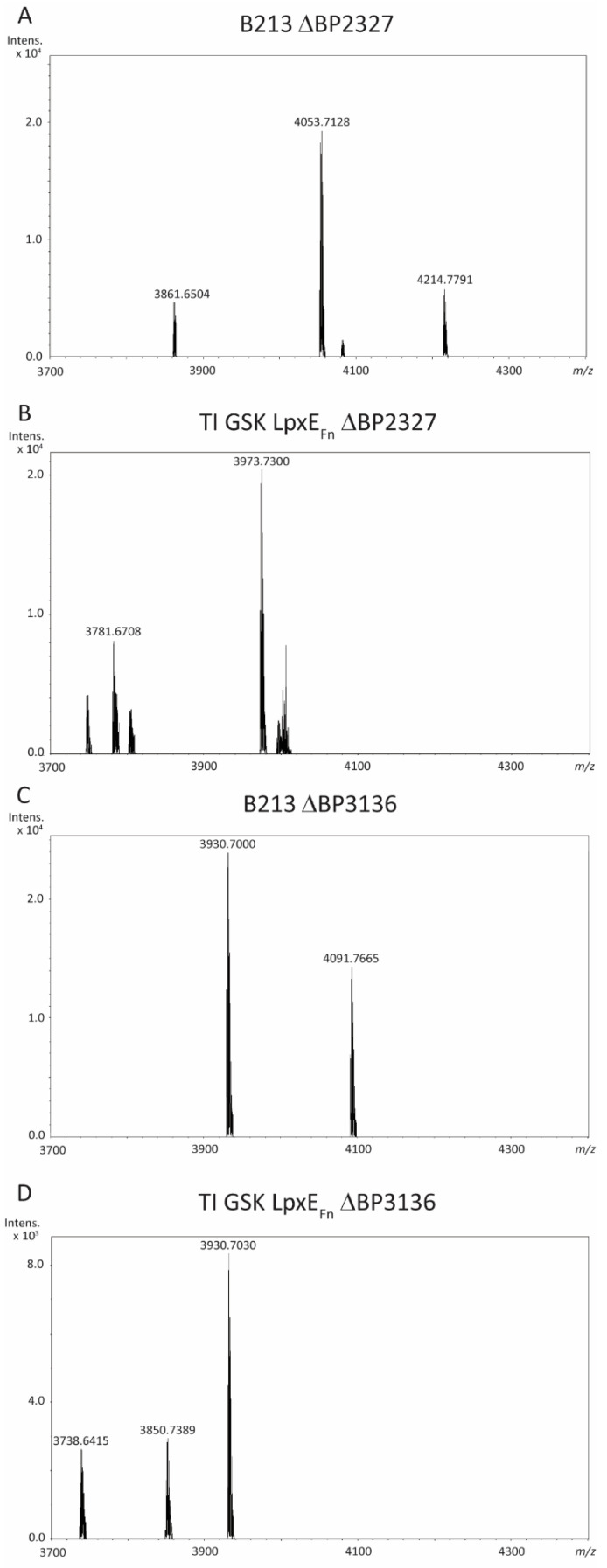
LC-MS analysis of LPS of *B. pertussis* BP2327 and BP3136 mutants. Shown are the spectra from B213 ΔBP2327 (**A**), TI GSK LpxE_Fn_ ΔBP2327 (**B**), B213 ΔBP3136 (**C**), and TI GSK LpxE_Fn_ ΔBP3136 (**D**). The measured monoisotopic mass of the peaks characterized is indicated. The peak at *m*/*z* 3930.7 corresponds to the structure of wild-type LPS with loss of PEA decoration. For interpretation of the other peaks, see Table 1.

Both genes were inactivated in strains B213 and TI GSK LpxE_Fn_, and the LPS structure was analyzed using LC-MS. Upon inactivation of BP2327, no relevant differences in the spectrum were observed compared with the LPS of their parental strains (Table 1). Thus, for B213 ΔBP2327, peaks corresponding to the main wild-type LPS species (*m*/*z* 4053.7) and either loss of heptose (*m*/*z* 3861.7) or inclusion of GlcN decoration (*m*/*z* 4214.8) were obtained (Figure 8A), whilst the spectrum for strain TI GSK LpxE_Fn_ ΔBP2327 showed peaks at *m*/*z* 3973.7 and 3781.7 (Figure 8B), which correspond to the dephosphorylated forms of *m*/*z* 4053.7 and 3861.7, respectively. In contrast, inactivation of BP3136 in strain B213 resulted in LPS forms at *m*/*z* 3930.7 and 4091.8 (Figure 8C), which correspond to the molecular ions at *m*/*z* 4053.7 and 4214.8, respectively, with the loss of the PEA decoration. The LPS of strain TI GSK LpxE_Fn_ ΔBP3136 showed peaks at *m*/*z* 3930.7 and 3738.6 (Figure 8D), which represent LPS forms derived from the wild-type LPS species with the loss of PEA and the same structure with the additional loss of heptose, respectively (Table 1). In these LPS species, dephosphorylation by LpxE_Fn_ had not taken place. An additional peak at *m*/*z* 3850.7, however, corresponds to the dephosphorylated form of *m*/*z* 3930.7 (Figure 8D, Table 1). These results demonstrate that BP3136 encodes the EptB protein that decorates the phosphate on Kdo with PEA and that LpxE_Fn_ activity in *B. pertussis* is reduced in the absence of the PEA decoration.

### 2.5. TLR4 Activation by Inner-Core-Modified Derivatives of B. pertussis

**Figure 9:**
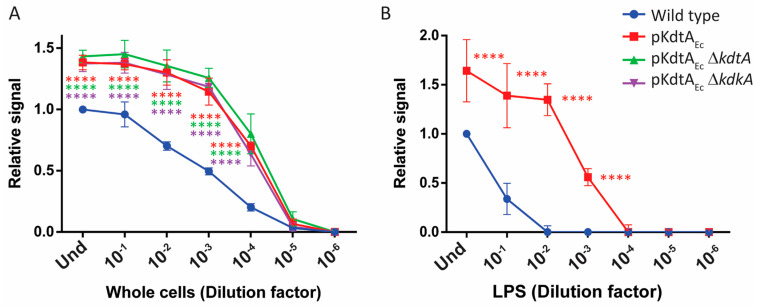
TLR4 activation by whole-cell preparations and purified LPS of strain B213 (wild type) and its pKdtA_Ec_-containing derivatives. TLR4-expressing HEK-Blue cells were incubated for 17 h with 10-fold serial dilutions of either heat-inactivated whole cells, where the OD_600_ of the undiluted suspensions (Und) was 0.15 (**A**), or purified LPS (Und: 20 nmol heptose/mL) (**B**). Graphs show means and standard deviations of relative SEAP activity calculated as the ratio between the signal measured for each dilution with each strain or LPS preparation and the signal measured for the Und parental strain or LPS preparation. Three independent experiments were performed in duplicate (whole cells) or singularly (LPS). Dilutions of the mutants with activity levels statistically different from those of the parental strain are indicated with asterisks (**** *p* ≤ 0.0001).

The effect of KdtA_Ec_ production on TLR4-stimulating activity was tested with whole-cell preparations of B213 and derivatives. All the recombinant strains showed significantly higher TLR4-stimulating activity than the wild type, with around 100-fold higher concentrations of wild-type suspension being required to reach a similar signal compared with the mutants (Figure 9A). Comparable results were obtained with the KdtA_Ec_-producing derivatives from strain TI GSK LpxE_Fn_ (Figure S7).

The increased TLR4-stimulating activity of the cells could be a direct effect of the modifications in the LPS structure on TLR4 recognition or a consequence of the unstable anchoring of LPS on the cell surface resulting in increased LPS release and availability for the receptor [9]. To discriminate between these possibilities, purified LPS preparations were also tested. TLR4 was significantly more strongly activated with LPS from the KdtA_Ec_-producing derivative than with LPS from the wild type (Figure 9B), confirming the effect of the structural alterations in LPS on TLR4 activation.

**Figure 10:**
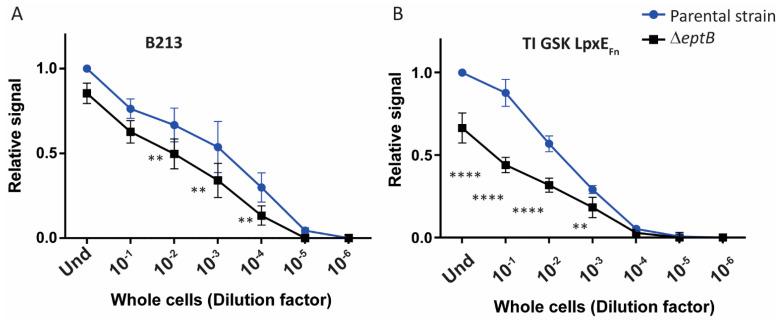
TLR4 activation by whole-cell preparations of strain B213 (**A**) and strain TI GSK LpxE_Fn_ (**B**) (Parental strains) and their *eptB* mutant derivatives. TLR4-expressing HEK-Blue cells were incubated for 17 h with 10-fold serial dilutions of heat-inactivated whole cells. The OD_600_ of the undiluted suspensions (Und) was 0.15. Graphs show means and standard deviations of relative SEAP activity calculated as the ratio between the signal measured for each dilution with each strain and the signal measured for the Und parental strain. Three independent experiments were performed in duplicate. Dilutions of the mutant with activity levels statistically different from those of the parental strain are indicated with asterisks (** *p* ≤ 0.01; **** *p* ≤ 0.0001).

The possible effect of the loss of the PEA group on TLR4 signaling was also studied. Whole cells of the *eptB* mutant of B213 had a significantly reduced ability to activate TLR4 (Figure 10A). This reduction was even more prominent in strain TI GSK LpxE_Fn_ (Figure 10B), which might be the consequence of the combined loss of PEA and lipid A-bound phosphate in a portion of the LPS produced by this strain (Figure 8D).

## 3. Discussion

Dephosphorylation is generally known to reduce the TLR4-stimulating activity of lipid A [34]. After synthesis of the lipid A phosphatases LpxE_Fn_ and LpxF_Fn_ in *B. pertussis*, only the production of LpxE_Fn_ resulted in the loss of a phosphate. Interestingly, despite the very efficient removal of the 1-phosphate upon expression of LpxE_Fn_, TLR4 activation by whole-cell preparations was unaffected. This could be related to the presence of phosphorylated Kdo in the inner core of *B. pertussis* LPS, which might functionally replace the lipid A 1-phosphate in the interaction with the TLR4–MD2 receptor complex. In a previous study in *C. canimorsus*, the importance of the core in TLR4 activation was shown [17]. In contrast to *E. coli* lipid A, the purified lipid A of *C. canimorsus*, which is dephosphorylated at position 4′ and presents PEA instead of phosphate at position 1, was unable to activate the receptor. However, the lipid A core fraction of this LPS was active. The authors hypothesized that the negative charge of the carboxyl group of the single Kdo in the core region facilitates the binding of LPS to MD2 and, thereby, TLR4 activation.

We hypothesized that modifications in the inner core could similarly affect TLR4 activation in *B. pertussis*. Our attempts to inactivate the *kdtA* and *kdkA* genes were futile, which is in line with their previously predicted essentiality [35,36]. Nevertheless, we succeeded in inactivating these genes in a recombinant strain producing the bi-functional KdtA of *E. coli*. Apparently, the presence of a Kdo substituted with either phosphate or a second Kdo in the inner core is essential for the viability of *B. pertussis*. This requirement seems to be similar for *E. coli*, whose bi-functional KdtA could only be replaced with a mono-functional homolog when a *kdkA* gene was co-expressed and, thereby, the single Kdo was phosphorylated [37]. Interestingly, the presence of KdoII in *B. pertussis* LPS appeared to impair the substitution of the outer core with the terminal trisaccharide. Differences in the core structure due to the presence of either a phosphate or a second Kdo attached to KdoI naturally occur in other species, such as *Pasteurella multocida* [38]. The depletion of the trisaccharide in *B. pertussis* producing KdtA_Ec_ could be due to the KdoII-mediated steric hindrance of the transferase that attaches the terminal trisaccharide. This transferase has not been characterized yet but is likely encoded by the *wlbI* gene [24,39]. Another possibility is that the phosphate group normally attached to KdoI facilitates the activity of the transferase. Alternatively, the loss of the terminal trisaccharide could be the result of the partial switch to the Bvg^−^ phase. The lack of band A, i.e., the absence of the trisaccharide, has been reported in *bvg* mutant strains [40,41]. We observed a switch of Bvg phase in some of the mutants containing KdoII, although all the Kdo-modified strains showed reduced amounts of trisaccharides. However, according to the SDS-PAGE analysis, there is a strong correlation between the extent of the loss of trisaccharides (Figure 3 and Figure S2) and the observed Bvg switch (Figure 4 and Figure S5). Despite this plausible connection, other factors may also be implicated, as suggested by a previous study in which the author could not directly associate the Bvg phase with the presence or absence of the trisaccharide [42]. The situation in *B. pertussis* is reminiscent of that in *E. coli*, where the Kdo disaccharide in the inner core can be decorated with a third Kdo, PEA, or rhamnose. These decorations affect the substitution of the outer core with a disaccharide [43]. Kdo decoration and attachment of the terminal disaccharide in *E. coli* are controlled by a complex regulatory network that involves the two-component system PhoP/Q, which is similar to the BvgA/S system of *B. pertussis* [44], as well as the RpoE-dependent envelope stress response and the two-component systems PhoB/R and BasS/R [43].

In the KdoII-containing strains, variable loss of the secondary C_14_ chain at the 2′ position of lipid A was also observed (Figure 5A,B). This acyl chain is inserted by the acylase LpxL2 [45]. The *B. pertussis* LpxL2 enzyme was previously shown, in vitro, to be optimally active on substrates with phosphorylated Kdo in the inner core [46]. The presence of KdoII instead of the phosphate decoration reduced but did not fully prevent its activity [46], which explains why LPS molecules with a secondary C_14_ chain were still detected in the spectra (Figure 5A,B) and why the strains were viable even though the *lpxL2* gene and thus the secondary C_14_ chain are essential for viability [35]. Nevertheless, the growth reduction observed in the KdoII-containing strains (Figure S3) might reflect the reduced incorporation of this acyl chain. Moreover, defects in cell division, which most likely reflect cellular stress caused by underacylated LPS, were observed (Figure 7). The presence of KdoII in *B. pertussis* LPS also prevented lipid A dephosphorylation in the strain producing LpxE_Fn_, perhaps due to steric hindrance. However, this contrasts with the situation in *F. novicida*, where LpxE_Fn_ dephosphorylates lipid A in the presence of KdoII [12].

The gene responsible for decorating the KdoI-linked phosphate with PEA, i.e., *eptB*, has hitherto not been identified in *B. pertussis*. We selected two possible candidates for *eptB*. Inactivation of BP2327, which shows homology to the *eptA* of other Gram-negative bacteria, did not have any effect on the LPS structure (Figure 8A), even though the gene resides in a cluster of genes involved in core biosynthesis [31]. Inactivation of BP3136, which shows similarity to the *eptB* gene of other species [32,33], however, resulted in the loss of the PEA substituent. Interestingly, like in the strains decorated with KdoII on KdoI, lipid A dephosphorylation was diminished upon inactivation of *eptB* in the strain producing LpxE_Fn_. Apparently, the presence of PEA is a prerequisite for the optimal activity of the phosphatase on *B. pertussis* LPS. On the other hand, production of the 4′-phosphatase LpxF_Fn_ in *B. pertussis* had no impact on the LPS structure. The inability of LpxF_Fn_ to dephosphorylate hexa-acylated LPS has previously been documented [13]. However, its activity on penta-acylated molecules, like that present in *B. pertussis*, is commonly observed. The LpxF of *H. pylori* is hindered by the presence of KdoII [47] and could thus also be hindered by the PEA decoration of KdoI in *B. pertussis*. However, this does not seem to be the case for LpxF_Fn_, which has been shown to be active in the absence of Kdo, as well as in the presence of either one or two Kdo residues [13,48], discarding any influence of other core features on LpxF_Fn_ activity. Nevertheless, it could be that the phosphatase encounters a physical impediment in the wild-type LPS of *B. pertussis* similar but opposite to that described for LpxE_Fn_. While KdoII-modified LPS might interfere with the access of LpxE_Fn_ to the 1-phosphate, the wild-type LPS organization might instead block access to the 4′-phosphate. If this hypothesis is correct, the production of LpxF_Fn_ in the *eptB* mutant could possibly facilitate 4′-dephosphorylation. This modification could affect TLR4 activation and is a strategy that could be considered in the future.

The production of KdtA_Ec_ increased the TLR4-activating capacity of *B. pertussis* cells. In contrast, the inactivation of *eptB* reduced TLR4 activation, and the inactivation of *eptB* in the LpxE_Fn_-producing strain reduced the TLR4-stimulating capacity even further, even though the phosphatase activity of LpxE_Fn_ was limited by the *eptB* mutation. Thus, *eptB* inactivation, especially in combination with LpxE_Fn_ production, seems a promising genetic approach to the development of novel whole-cell vaccines against pertussis with reduced endotoxicity, and it is worthwhile to further investigate the immunogenicity and reactogenicity of such vaccines in vivo.

Overall, we describe the *eptB* gene in *B. pertussis* and show that the PEA substitution attached by the encoded enzyme to the Kdo-linked phosphate is involved in TLR4 activation. Furthermore, the insertion of a second Kdo in the inner core affects the structure of the outer core by inhibiting the attachment of the terminal trisaccharide and of lipid A by limiting the attachment of a secondary acyl chain. These modifications result in defective cell division and, surprisingly, considering the partial loss of an acyl chain, in an increased TLR4-stimulating capacity of these cells. In addition, the lipid A dephosphorylation accomplished through LpxE_Fn_ production, which shows no effect on TLR4 stimulation on its own, is abolished through the introduction of a second Kdo and remarkably reduced by the absence of the inner-core PEA. To our knowledge, this is the first study focusing on structural modifications in the inner core of *B. pertussis* and their effect on endotoxicity.

## 4. Materials and Methods

### 4.1. Bacterial Strains and Growth Conditions

All bacterial strains used are described in Table S1. *E. coli* was grown at 37 °C in lysogeny broth (LB) while shaking or on LB agar plates. *B. pertussis* was grown at 35 °C on Bordet–Gengou agar (Difco) supplemented with 15% defibrinated sheep blood (Biotrading, Mijdrecht, The Netherlands) (BG-blood). For liquid cultures, bacteria were scraped from BG-blood plates, on which they had been growing for three days; inoculated in Verwey medium [49] to an optical density at 600 nm (OD_600_) of 0.05; and grown for 24 h at 35 °C while shaking at 175 rpm. When appropriate, the media were supplemented with nalidixic acid (50 µg/mL), streptomycin (300 µg/mL), gentamicin (10 µg/mL), kanamycin (50 µg/mL), and/or ampicillin (100 µg/mL) for plasmid maintenance or strain selection. To induce gene expression from plasmids, 1 mM isopropyl-β-D-thiogalactopyranoside (IPTG) was added to the medium.

### 4.2. DNA Manipulation and Plasmid Construction

All the plasmids and relevant PCR primers used are listed in Tables S1 and S2, respectively. Regular PCR reactions were performed using DreamTaq DNA polymerase (Thermo Scientific, Waltham, MA, USA), whilst PCR fragments generated for cloning were obtained using the Expand High Fidelity PCR system (Roche Diagnostics GmbH, Indianapolis, IN, USA). For the purification of PCR products, the commercial Wizard SV Gel and PCR Clean-Up System (Promega, Madison, WI, USA) was employed. Plasmids were isolated with E.Z.N.A. Plasmid Mini Kit I (Omega Bio-Tek, Norcross, GA, USA). PCR products and plasmids were digested with the appropriate restriction enzymes according to manufacturer’s instructions (Thermo Scientific), purified, and ligated using T4 DNA ligase (5 U/µL) (Thermo Scientific). All plasmids constructed were verified using DNA sequencing (Macrogen, Seoul, Republic of Korea), and their presence in the selected clones was confirmed using PCR.

To express the *lpxE_Fn_* (GenBank accession number AY713119) and *lpxF_Fn_* (GenBank accession number DQ364143) genes in *B. pertussis*, codon-optimized sequences of these genes were synthesized (PriorityGENE service, GENEWIZ, Azenta Life Sciences, Burlington, MA, USA). The *lpxF_Fn_* synthetic fragment included Acc65I and HindIII restriction sites on the flanks and was inserted in pMMB67EH through Acc65I-HindIII digestion and subsequent ligation. The codon-optimized *lpxE_Fn_* fragment was amplified using the forward primer Fw-XbaI-LpxE, which introduces an XbaI restriction site at the 5′ end of the gene, and the reverse primer Rv-LpxE-ApaI, which introduces an ApaI restriction site at the 3′ end. The resulting fragment was then cloned using these restriction sites into a pUC19-based plasmid containing *lgmB* upstream and downstream sequences flanking the *ompP* (BP0840) promoter region followed by XbaI and ApaI sites (sequences from *B. pertussis* genome sequence, accession number CP039022.1; plasmid provided by Gianmarco Gasperini, GSK). The resulting construct was subcloned in pSORTP1 using the EcoRI and HindIII restriction sites from the pUC19 multiple cloning site. *E. coli* strain DH5α was transformed with the pMMB67EH and pSORTP1 constructs using electroporation. Plasmid DNA was isolated from transformants and introduced in *E. coli* strain SM10λ*pir* for conjugation into *B. pertussis*. For pMMB67EH, transconjugants were selected on plates containing ampicillin for plasmid selection and nalidixic acid for counterselection against *E. coli*. For integration of the pSORPT1 construct into the *B. pertussis* genome, transconjugants were selected on plates containing gentamicin for plasmid selection and nalidixic acid for counterselection against *E. coli*. Subsequently, the removal of the plasmid backbone was selected by cultivation on plates containing streptomycin. The presence of the *lpxE_Fn_* sequence inside the genomic *lgmB* locus was confirmed using PCR and DNA sequencing.

The *kdtA_Ec_* gene (locus tag JW3608) was PCR-amplified from *E. coli* strain W3110 using the forward primer Fw-XbaI-KdtA-Ec, which introduces an XbaI restriction site upstream of the gene, and the reverse primer Rv-KdtA-His-RBS-NdeI-HindIII, which introduces a DNA segment encoding a C-terminal His-Tag, a ribosome-binding site, an NdeI restriction site (the latter two were included for future co-expression of other genes), and a HindIII restriction site at the 3′ end of the gene. The amplified fragment was inserted in pMMB67EH through XbaI-HindIII digestion and subsequent ligation. The resulting plasmid was used to transform *E. coli* strain DH5α using the CaCl_2_ method. Plasmid DNA was extracted from transformants and introduced in *E. coli* strain SM10λ*pir*, which allowed for transfer to *B. pertussis* through conjugation as described above. Expression was confirmed using Western blotting with anti-His antibodies.

To inactivate the *kdtA* (locus tag BP0095), *kdkA* (locus tag BP2349), BP2327, and BP3136 (*eptB*) genes of *B. pertussis*, synthetic DNA fragments that included 800 bp fragments upstream and downstream of the target genes in reference strain Tohama I were designed. The entire sequence of each target gene, except for bp overlapping with other genes, was replaced in the synthetic sequence with a gentamicin-resistance (*gem^R^*) cassette flanked by Eco81I restriction sites. In addition, we arranged the complete synthetic fragments so that they were flanked by XbaI restriction sites. The artificial sequences were introduced in pUC57-Kan through blunt-end ligation into the SmaI restriction site of the vector (services provided by BaseGene, Leiden, The Netherlands). The plasmids were used to transform *E. coli* strain DH5α. Strain selection was performed on LB plates containing either kanamycin or a combination of kanamycin and gentamicin. No growth was observed on the plates with both antibiotics, suggesting that the *gem^R^* cassette was not expressed. Therefore, another *gem^R^* cassette was PCR-amplified from plasmid pYRC using the primers Fw-Eco81I-GemR (forward) and Rv-GemR-Eco81I (reverse), which introduced, on both flanks, Eco81I restriction sites. This amplicon was used to replace the synthetic *gem^R^* cassette through digestion of the plasmid and PCR product with Eco81I and subsequent ligation. Transformants of DH5α were obtained on LB plates containing the combination of both antibiotics, and the orientation of the cassette in the direction of the transcription of the operon of the target genes was confirmed using PCR on isolated colonies. The knockout construct obtained was then subcloned into the suicide vector pKAS32 through XbaI digestion and ligation. The resulting plasmids were used to transform *E. coli* strain SM10λ*pir* and subsequently transferred to *B. pertussis* through conjugation. Chromosomal knockouts were obtained through allelic exchange. First, transconjugants were selected on plates containing gentamicin for selection and nalidixic acid for counterselection against *E. coli*. Subsequently, selection was made with streptomycin and gentamicin to ensure the loss of the plasmid backbone together with the wild-type gene.

### 4.3. LC-MS

Bacterial cultures, grown for 24 h, were centrifuged at 5000× *g* for 15 min at 4 °C, and the resulting pellet was resuspended in 10 mL of purification buffer (100 mM Tris-HCl, 10 mM EDTA, pH 8.6). Then, the cells were inactivated for 30 min at 56 °C and sonicated for 20 min in cool water. After treatment with Benzonase Nuclease (50 U/mL; Millipore, Burlington, MA, USA) for 1 h, unbroken cells and aggregates were removed using centrifugation for 30 min at 20,000× *g* and 4 °C. To the supernatant, purification buffer was added up to 16.5 mL, and the resulting suspension was centrifuged at 40,000 rpm for 2 h at 4 °C (Beckman Coulter Optima LE-80K, Type 70 Ti rotor; Brea, CA, USA). The resulting pellet was resuspended in PBS.

The resuspended pellet was subjected to ethanol precipitation by adding 300 μL of 95% ethanol, which was precooled at −20 °C, to 100 μL of sample; vortexing; and incubation for 1 h at −20 °C. Then, the samples were centrifuged at 13,000 rpm for 15 min at 4 °C, and the pellet was dissolved in 50 μL of 50% (*v*/*v*) aqueous methanol solution. After subjecting the samples to vortexing and an ultrasonic bath for 10 min, they were further dissolved with an ultrasonic microprobe for 1 min and centrifuged at 13,000 rpm for 10 min at 4 °C. For LC-MS analysis in negative-ion mode, the supernatant was injected in a Thermofisher Scientific Dionex U3000 RSLC with an Acquity UPLC BEH130 C18 (either 1 mm × 50 mm, 1.7 μm or 300 μm × 50 mm, 1.7 μm) column and analyzed as previously described [50]. All mass spectra reported are monocharged ([M-H]^−^) and deconvoluted.

### 4.4. OM Isolation

OMs were isolated as previously described [51]. Briefly, the bacterial pellet collected from liquid cultures was resuspended to an OD_600_ of 7.5 in 2 mL of physiological salt solution and inactivated using incubation for 30 min at 56 °C. Then, cells were harvested, and spheroplasts were made with the addition of 2 mL of 0.75 M sucrose in 10 mM Tris-HCl (pH 7.8), 10 µL of 40 mg/mL lysozyme, and 4 mL of 1.5 mM EDTA (pH 7.5) [52]. After freezing the spheroplasts at −80 °C, they were lysed using ultrasonication and centrifuged at 10,000× *g* for 1 h at 4 °C. To collect the OM, the supernatant was centrifuged at 40,000 rpm for 1 h at 4 °C (Beckman Coulter Optima LE-80K, Type 70 Ti rotor), and the resulting pellet was resuspended in PBS.

### 4.5. Kdo Assay

Kdo content was quantified in isolated OMs as previously described [53] with slight modifications. Briefly, 50 µL of OM sample was mixed with 50 µL of 0.25 M H_2_SO_4_ and boiled for 20 min. After cooling down to room temperature, 50 µL of 0.1 M H_5_IO_6_ was added, and the mixture was incubated for 10 min before the addition of 200 µL of 4% (*w*/*v*) NaAsO_2_. After the yellow color vanished, 800 µL of 0.3% (*w*/*v*) thiobarbituric acid was added, and the mixture was boiled for 10 min. While the solution was still hot, 250 µL of DMSO was added, and the OD_550_ was measured immediately afterwards. Known concentrations of 2-keto-3-deoxyoctonate ammonium salt (Sigma-Aldrich, St. Louis, MO, USA) were used to plot a standard curve.

### 4.6. SDS-PAGE and Western Blotting

OM preparations were mixed with sample buffer [54], boiled for 10 min, and analyzed on Mini-PROTEAN TGX Precast Protein Gels (Bio-Rad, Hercules, CA, USA) containing either 10 or 4–15% polyacrylamide for protein or LPS analysis, respectively. After electrophoresis, proteins or LPS were stained with Bradford reagent [55] or with silver [56], respectively. Images of the relevant parts of the gels were taken using the Bio-Rad imaging system. Alternatively, the proteins were transferred from gel to a 0.45 μm pore-size nitrocellulose membrane (GE Healthcare, Chicago, IL, USA). Based on the positions of the molecular-weight standard proteins, the blots were usually cut into three pieces containing high-, middle-, and low-molecular-weight proteins, respectively, and these segments were separately incubated with mouse antiserum directed against autotransporter BrkA and rabbit antisera directed against the major porin OmpP and a synthetic peptide of the OM-associated protein RmpM [57], respectively. The antisera were used at dilutions of 1:4000, 1:80,000, and 1:5000, respectively. Horseradish peroxidase-conjugated goat anti-mouse or anti-rabbit IgG secondary antibodies (ThermoFisher) were employed at the dilution of 1:10,000. Membranes were developed with Clarity Western ECL Blotting Substrate (Bio-Rad).

### 4.7. Microscopy

Bacterial cultures were fixed with 1% formaldehyde for at least 30 min at 4 °C and stained for 15 min in the dark with either 5 µg/mL of FM4-64 (Invitrogen, Waltham, MA, USA) or a mixture of SYTO 9 (5 µM) and propidium iodide (30 µM) (Live/Dead BacLight kit; Invitrogen). Then, 5 µL samples of the stained suspensions were pipetted onto 1%-agarose pads placed on microscopy slides, and cells were visualized with a Zeiss Axioskop 2 fluorescence microscope (Zeiss, Jena, Germany) with a 100× objective.

### 4.8. LPS Isolation and Heptose Quantification

Bacterial cultures were adjusted to an OD_600_ of 0.05 in fresh medium and grown for another 24 h. Then, cells were pelleted for 10 min at 10,000× *g* and washed with Milli-Q water. After centrifugation, the washed pellet was resuspended in Milli-Q water and lyophilized. LPS were isolated from dry cells as described [58] with slight modifications. Briefly, extraction was performed using a solution of phenol/chloroform/petroleum ether (PCP; 2:5:8, *v*/*v*/*v*). After extraction with 1 mL of PCP per ~100 mg of dry cells and vortexing for 15 min, samples were centrifuged for 20 min at 10,000× *g*. Supernatants were collected, and chloroform ether and petroleum ether were evaporated using alternate incubation in a water bath at 50 °C and air stream, until the remaining phenol crystalized. Then, 1.5 mL of acetone was added per ml of PCP originally used. Precipitated LPS were collected using centrifugation for 20 min at 10,000× *g*. The resulting pellet was allowed to dry completely, and LPS were resuspended in Milli-Q water by alternating incubation at 60 °C and vortexing. LPS were quantified based on heptose content, which was determined with the cysteine–sulfuric acid method [59] as modified by Osborn [60].

### 4.9. TLR4 Stimulation Assays

TLR4 stimulation assays were performed as previously described [61]. Briefly, HEK-Blue TLR4 reporter cells co-expressing human TLR4, MD-2, and CD14 genes (Invivogen, San Diego, CA, USA) were incubated with serial dilutions of either whole bacterial cells that were killed using heat treatment at 56 °C for 30 min or isolated LPS. After 17 h of incubation at 37 °C in a 5%-saturated CO_2_ atmosphere, supernatants were incubated with *p*-nitrophenyl phosphate solution for 1 h, and the absorbance at 405 nm was measured with a Biotek microplate reader (Winooski, VT, USA).

### 4.10. Statistical Analysis

All statistical analyses were performed using GraphPad Prism software, version 6. TLR4 stimulation data were analyzed for statistical significance using two-way ANOVA (Dunnett’s correction for multiple comparisons) when more than two strains were compared, while the multiple *t*-test (Holm–Sidak correction) was used for the comparison of two strains. One-way ANOVA (Tukey’s correction for multiple comparisons) was performed on Kdo quantification studies.

## Figures and Tables

**Table 1:** Main ions detected in LC-MS data and proposed compositions of LPS of the *B. pertussis* strains analyzed in this study.

Figure	Strain	Measured Monoisotopic Mass [M-H]^−^	Calculated Monoisotopic Mass [M-H]^−^	Mass Error (ppm)	Proposed Composition
2A	Tohama I GSK	40,537,067	40,537,570	−12	Wt (TerTri ^a^∙ GalNA∙ Glc∙ GlcN_2_∙ GlcA∙ Hep_3_∙ PPEA∙ Kdo∙ lipid A)
		38,616,673	38,616,936	−7	Wt -Hep
		42,147,967	42,148,258	−7	Wt +GlcN
		33,924,523	33,924,764	−7	Wt -TerTri
2B	TI GSK LpxF_Fn_	40,537,248	40,537,570	−8	Wt
		38,616,625	38,616,936	−8	Wt -Hep
		42,147,915	42,148,258	−8	Wt +GlcN
2C	TI GSK LpxE_Fn_	39,737,829	39,737,907	−2	Wt-P
		37,817,040	37,817,273	−6	Wt -Hep -P
		40,537,363	40,537,570	−5	Wt
4A	B213	40,536,929	40,537,570	−16	Wt
		42,147,700	42,148,258	−13	Wt +GlcN
		38,616,441	38,616,936	−13	Wt -Hep
		33,924,317	33,924,764	−13	Wt -TerTri
4B	B213 pKdtA_Ec_ Δ*kdtA*	34,095,180	34,095,598	−12	Wt -PPEA -TerTri +Kdo
		40,707,891	40,708,405	−13	Wt -PPEA +Kdo
5A	TI GSK LpxE_Fn_ pKdtA_Ec_ Δ*kdtA*	34,095,187	34,095,598	−12	Wt -PPEA -TerTri +Kdo
		31,993,224	31,993,615	−12	Wt -PPEA -TerTri +Kdo -C14
		38,605,950	38,606,421	−12	Wt -PPEA +Kdo -C14
		40,707,889	40,708,405	−13	Wt -PPEA +Kdo
5B	TI GSK LpxE_Fn_ pKdtA_Ec_ Δ*kdkA*	34,095,183	34,095,598	−12	Wt -PPEA -TerTri +Kdo
		40,707,872	40,708,405	−13	Wt -PPEA +Kdo
		38,605,930	38,606,421	−13	Wt -PPEA +Kdo -C14
		31,993,227	31,993,615	−12	Wt -PPEA -TerTri +Kdo -C14
8A	B213 ΔBP2327	40,537,128	40,537,570	−11	Wt
		42,147,791	42,148,258	−11	Wt +GlcN
		38,616,504	38,616,936	−11	Wt -Hep
8B	TI GSK LpxE_Fn_ ΔBP2327	39,737,300	39,737,907	−15	Wt -P
		37,816,708	37,817,273	−15	Wt -Hep -P
8C	B213 ΔBP3136	39,307,000	39,307,485	−12	Wt -PEA
		40,917,665	40,918,173	−12	Wt +GlcN -PEA
8D	TI GSK LpxE_Fn_ ΔBP3136	39,307,030	39,307,485	−12	Wt -PEA
		38,507,389	38,507,822	−11	Wt -PEA -P
		37,386,415	37,386,851	−12	Wt -Hep -PEA

^a^ TerTri, terminal trisaccharide sugars (GlcNAc∙Man2NAc3NAcA∙Fuc2NAc4NMe).

## Data Availability

Data are contained within the article and its Supplementary Materials. Additional data supporting the findings of this study are available from the corresponding author upon reasonable request.

## Supplementary Materials

The following supporting information can be downloaded at: https://www.mdpi.com/article/10.3390/ijms242417313/s1. References [62,63,64,65,66,67,68] are cited in the Supplementary Materials.

## References

[1] Mattoo S., Cherry J.D. (2005). Molecular pathogenesis, epidemiology, and clinical manifestations of respiratory infections due to *Bordetella pertussis* and other *Bordetella* subspecies. Clin. Microbiol. Rev..

[2] Esposito S., Stefanelli P., Fry N.K., Fedele G., He Q., Paterson P., Tan T., Knuf M., Rodrigo C., Weil Olivier C. (2019). Pertussis prevention: Reasons for resurgence, and differences in the current acellular pertussis vaccines. Front. Immunol..

[3] Silhavy T.J., Kahne D., Walker S. (2010). The bacterial cell envelope. Cold Spring Harb. Perspect. Biol..

[4] Raetz C.R.H., Whitfield C. (2002). Lipopolysaccharide endotoxins. Annu. Rev. Biochem..

[5] Parrillo J.E. (1993). Pathogenetic mechanisms of septic shock. N. Engl. J. Med..

[6] Park B.S., Song D.H., Kim H.M., Choi B.S., Lee H., Lee J.O. (2009). The structural basis of lipopolysaccharide recognition by the TLR4–MD-2 complex. Nature.

[7] Raetz C.R.H., Reynolds C.M., Trent M.S., Bishop R.E. (2007). Lipid A modification systems in Gram-negative bacteria. Annu. Rev. Biochem..

[8] Matsuura M. (2013). Structural modifications of bacterial lipopolysaccharide that facilitate Gram-negative bacteria evasion of host innate immunity. Front. Immunol..

[9] Geurtsen J., Steeghs L., Hamstra H.J., ten Hove J., de Haan A., Kuipers B., Tommassen J., van der Ley P. (2006). Expression of the lipopolysaccharide-modifying enzymes PagP and PagL modulates the endotoxic activity of *Bordetella pertussis*. Infect. Immun..

[10] Arenas J., Pupo E., Phielix C., David D., Zariri A., Zamyatina A., Tommassen J., van der Ley P. (2020). Shortening the lipid A acyl chains of *Bordetella pertussis* enables depletion of lipopolysaccharide endotoxic activity. Vaccines.

[11] Marr N., Tirsoaga A., Blanot D., Fernandez R., Caroff M. (2008). Glucosamine found as a substituent of both phosphate groups in *Bordetella* lipid A backbones: Role of a BvgAS-activated ArnT ortholog. J. Bacteriol..

[12] Wang X., Karbarz M.J., McGrath S.C., Cotter R.J., Raetz C.R.H. (2004). MsbA transporter-dependent lipid A 1-dephosphorylation on the periplasmic surface of the inner membrane: Topography of *Francisella novicida* LpxE expressed in *Escherichia coli*. J. Biol. Chem..

[13] Wang X., McGrath S.C., Cotter R.J., Raetz C.R.H. (2006). Expression cloning and periplasmic orientation of the *Francisella novicida* lipid A 4′-phosphatase LpxF. J. Biol. Chem..

[14] Kong Q., Six D.A., Roland K.L., Liu Q., Gu L., Reynolds C.M., Wang X., Raetz C.R.H., Curtiss R. (2011). *Salmonella* synthesizing 1-dephosphorylated lipopolysaccharide exhibits low endotoxic activity while retaining its immunogenicity. J. Immunol..

[15] Simpson B.W., Trent M.S. (2019). Pushing the envelope: LPS modifications and their consequences. Nat. Rev. Microbiol..

[16] Kawahara K. (2021). Variation, modification and engineering of lipid A in endotoxin of Gram-negative bacteria. Int. J. Mol. Sci..

[17] Ittig S., Lindner B., Stenta M., Manfredi P., Zdorovenko E., Knirel Y.A., dal Peraro M., Cornelis G.R., Zähringer U. (2012). The lipopolysaccharide from *Capnocytophaga canimorsus* reveals an unexpected role of the core-oligosaccharide in MD-2 binding. PLoS Pathog..

[18] Caroff M., Aussel L., Zarrouk H., Martin A., Richards J.C., Thérisod H., Perry M.B., Karibian D. (2001). Structural variability and originality of the *Bordetella* endotoxins. J. Endotoxin Res..

[19] Caroff M., Brisson J.R., Martin A., Karibian D. (2000). Structure of the *Bordetella pertussis* 1414 endotoxin. FEBS Lett..

[20] White K.A., Lin S., Cotter R.J., Raetz C.R.H. (1999). A *Haemophilus influenzae* gene that encodes a membrane bound 3-deoxy-d-*manno*-octulosonic acid (Kdo) kinase: Possible involvement of Kdo phosphorylation in bacterial virulence. J. Biol. Chem..

[21] Reynolds C.M., Kalb S.R., Cotter R.J., Raetz C.R.H. (2005). A phosphoethanolamine transferase specific for the outer 3-deoxy-D-*manno*-octulosonic acid residue of *Escherichia coli* lipopolysaccharide: Identification of the *eptB* gene and Ca^2+^ hypersensitivity of an *eptB* deletion mutant. J. Biol. Chem..

[22] Isobe T., White K.A., Allen A.G., Peacock M., Raetz C.R.H., Maskell D.J. (1999). *Bordetella pertussis waaA* encodes a monofunctional 2-keto-3-deoxy-D- *manno*-octulosonic acid transferase that can complement an *Escherichia coli waaA* mutation. J. Bacteriol..

[23] Belunis C.J., Raetz C.R.H. (1992). Biosynthesis of endotoxins. Purification and catalytic properties of 3-deoxy-D-*manno*-octulosonic acid transferase from *Escherichia coli*. J. Biol. Chem..

[24] Allen A., Maskell D. (1996). The identification, cloning and mutagenesis of a genetic locus required for lipopolysaccharide biosynthesis in *Bordetella pertussis*. Mol. Microbiol..

[25] Allen A.G., Thomas R.M., Cadisch J.T., Maskell D.J. (1998). Molecular and functional analysis of the lipopolysaccharide biosynthesis locus *wlb* from *Bordetella pertussis*, *Bordetella parapertussis* and *Bordetella bronchiseptica*. Mol. Microbiol..

[26] Caroff M., Deprun C., Richards J.C., Karibian D. (1994). Structural characterization of the lipid A of *Bordetella pertussis* 1414 endotoxin. J. Bacteriol..

[27] Hamstra H.J., Kuipers B., Schijf-Evers D., Loggen H.G., Poolman J.T. (1995). The purification and protective capacity of *Bordetella pertussis* outer membrane proteins. Vaccine.

[28] Passerini de Rossi B.N., Friedman L.E., González Flecha F.L., Castello P.R., Franco M.A., Rossi J.P.F.C. (1999). Identification of *Bordetella pertussis* virulence-associated outer membrane proteins. FEMS Microbiol. Lett..

[29] de Jonge E.F., van Boxtel R., Balhuizen M.D., Haagsman H.P., Tommassen J. (2022). Pal depletion results in hypervesiculation and affects cell morphology and outer-membrane lipid asymmetry in bordetellae. Res. Microbiol..

[30] Weiss A.A., Hewlett E.L., Myers G.A., Falkow S. (1983). Tn*5*-induced mutations affecting virulence factors of *Bordetella pertussis*. Infect. Immun..

[31] Geurtsen J., Dzieciatkowska M., Steeghs L., Hamstra H.J., Boleij J., Broen K., Akkerman G., El Hassan H., Li J., Richards J.C. (2009). Identification of a novel lipopolysaccharide core biosynthesis gene cluster in *Bordetella pertussis*, and influence of core structure and lipid A glucosamine substitution on endotoxic activity. Infect. Immun..

[32] de Gouw D., Hermans P.W.M., Bootsma H.J., Zomer A., Heuvelman K., Diavatopoulos D.A., Mooi F.R. (2014). Differentially expressed genes in *Bordetella pertussis* strains belonging to a lineage which recently spread globally. PLoS ONE.

[33] Harper M., Wright A., St Michael F., Li J., Lucas D.D., Ford M., Adler B., Cox A.D., Boyce J.D. (2017). Characterization of two novel lipopolysaccharide phosphoethanolamine transferases in *Pasteurella multocida* and their role in resistance to cathelicidin-2. Infect. Immun..

[34] Maeshima N., Fernandez R.C. (2013). Recognition of lipid A variants by the TLR4-MD-2 receptor complex. Front. Cell Infect. Microbiol..

[35] Gonyar L.A., Gelbach P.E., McDuffie D.G., Koeppel A.F., Chen Q., Lee G., Temple L.M., Stibitz S., Hewlett E.L., Papin J.A. (2019). In vivo gene essentiality and metabolism in *Bordetella pertussis*. mSphere.

[36] Belcher T., MacArthur I., King J.D., Langridge G.C., Mayho M., Parkhill J., Preston A. (2020). Fundamental differences in physiology of *Bordetella pertussis* dependent on the two-component system Bvg revealed by gene essentiality studies. Microb. Genom..

[37] Brabetz W., Müller-Loennies S., Brade H. (2000). 3-Deoxy-D-*manno*-oct-2-ulosonic acid (Kdo) transferase (WaaA) and Kdo kinase (KdkA) of *Haemophilus influenzae* are both required to complement a *waaA* knockout mutation of *Escherichia coli*. J. Biol. Chem..

[38] Harper M., Boyce J.D., Cox A.D., Michael F.S., Wilkie I.W., Blackall P.J., Adler B. (2007). *Pasteurella multocida* expresses two lipopolysaccharide glycoforms simultaneously, but only a single form is required for virulence: Identification of two acceptor-specific heptosyl I transferases. Infect. Immun..

[39] Preston A., Thomas R., Maskell D.J. (2002). Mutational analysis of the *Bordetella pertussis wlb* LPS biosynthesis locus. Microb. Pathog..

[40] Schaeffer L.M., McCormack F.X., Wu H., Weiss A.A. (2004). *Bordetella pertussis* lipopolysaccharide resists the bactericidal effects of pulmonary surfactant protein A. J. Immunol..

[41] Turcotte M.L., Martin D., Brodeur B.R., Peppler M.S. (1997). Tn*5*-induced lipopolysaccharide mutations in *Bordetella pertussis* that affect outer membrane function. Microbiology.

[42] Van den Akker W.M.R. (1998). Lipopolysaccharide expression within the genus *Bordetella*: Influence of temperature and phase variation. Microbiology.

[43] Klein G., Raina S. (2015). Regulated control of the assembly and diversity of LPS by noncoding sRNAs. Biomed. Res. Int..

[44] Park S.Y., Groisman E.A. (2014). Signal-specific temporal response by the *Salmonella* PhoP/PhoQ regulatory system. Mol. Microbiol..

[45] Geurtsen J., Angevaare E., Janssen M., Hamstra H.J., ten Hove J., de Haan A., Tommassen J., van der Ley P. (2007). A novel secondary acyl chain in the lipopolysaccharide of *Bordetella pertussis* required for efficient infection of human macrophages. J. Biol. Chem..

[46] Hankins J.V., Trent S.M. (2009). Secondary acylation of *Vibrio cholerae* lipopolysaccharide requires phosphorylation of Kdo. J. Biol. Chem..

[47] Cullen T.W., Giles D.K., Wolf L.N., Ecobichon C., Boneca I.G., Trent M.S. (2011). *Helicobacter pylori* versus the host: Remodeling of the bacterial outer membrane is required for survival in the gastric mucosa. PLoS Pathog..

[48] Okan N.A., Chalabaev S., Kim T.H., Fink A., Ross R.A., Kasper D.L. (2013). Kdo hydrolase is required for *Francisella tularensis* virulence and evasion of TLR2-mediated innate immunity. mBio.

[49] Verwey W.F., Thiele E.H., Sage D.N., Schuchardt L.F. (1949). A simplified liquid culture medium for the growth of *Hemophilus pertussis*. J. Bacteriol..

[50] O’Brien J.P., Needham B.D., Brown D.B., Trent M.S., Brodbelt J.S. (2014). Top-down strategies for the structural elucidation of intact Gram-negative bacterial endotoxins. Chem. Sci..

[51] Pérez-Ortega J., van Boxtel R., de Jonge E.F., Tommassen J. (2022). Regulated expression of *lpxC* allows for reduction of endotoxicity in *Bordetella pertussis*. Int. J. Mol. Sci..

[52] Osborn M.J., Gander J.E., Parisi E., Carson J. (1972). Mechanism of assembly of the outer membrane of *Salmonella typhimurium*. Isolation and characterization of the cytoplasmic and outer membrane. J. Biol. Chem..

[53] Sunayana M.R., Reddy M. (2015). Determination of Keto-deoxy-d-manno-8-octanoic acid (KDO) from lipopolysaccharide of *Escherichia coli*. Bio-Protocol.

[54] Laemmli U. (1970). Cleavage of structural proteins during the assembly of the head of bacteriophage T4. Nature.

[55] Bos M.P., Tommassen-van Boxtel R., Tommassen J. (2015). Experimental methods for studying the BAM complex in *Neisseria meningitidis*. Methods Mol. Biol..

[56] Tsai C.M., Frasch C.E. (1982). A sensitive silver stain for detecting lipopolysaccharides in polyacrylamide gels. Anal. Biochem..

[57] de Jonge E.F., Balhuizen M.D., van Boxtel R., Wu J., Haagsman H.P., Tommassen J. (2021). Heat shock enhances outer-membrane vesicle release in *Bordetella* spp.. Curr. Res. Microb. Sci..

[58] Galanos C., Lüderitz O., Westphal O. (1969). A new method for the extraction of R lipopolysaccharides. Eur. J. Biochem..

[59] Dische Z. (1953). Qualitative and quantitative colorimetric determination of heptoses. J. Biol. Chem..

[60] Osborn M.J. (1963). Studies on the Gram-negative cell wall, I. Evidence for the role of 2-keto-3-deoxyoctonate in the lipopolysaccharide of *Salmonella* Typhimurium. Proc. Natl. Acad. Sci. USA.

[61] Pérez-Ortega J., van Harten R.M., van Boxtel R., Plisnier M., Louckx M., Ingels D., Haagsman H.P., Tommassen J. (2021). Reduction of endotoxicity in *Bordetella bronchiseptica* by lipid A engineering: Characterization of *lpxL1* and *pagP* mutants. Virulence.

[62] King A.J., Berbers G., van Oirschot H.F.L.M., Hoogerhout P., Knipping K., Mooi F.R. (2001). Role of the polymorphic region 1 of the *Bordetella pertussis* protein pertactin in immunity. Microbiology.

[63] Grant S.G.N., Jessee J., Bloom F.R., Hanahan D. (1990). Differential plasmid rescue from transgenic mouse DNAs into *Escherichia coli* methylation-restriction mutants. Proc. Natl. Acad. Sci. USA.

[64] Simon R., Priefer U., Pühler A. (1983). A broad host range mobilization system for in vivo genetic engineering: Transposon mutagenesis in Gram-negative bacteria. Nat. Biotechnol..

[65] Arts J., van Boxtel R., Filloux A., Tommassen J., Koster M. (2007). Export of the pseudopilin XcpT of the *Pseudomonas aeruginosa* type II secretion system via the signal recognition particle-Sec pathway. J. Bacteriol..

[66] Skorupski K., Taylor R.K. (1996). Positive selection vectors for allelic exchange. Gene.

[67] Stibitz S., Black W., Falkow S. (1986). The construction of a cloning vector designed for gene replacement in *Bordetella pertussis*. Gene.

[68] Fürste J.P., Pansegrau W., Frank R., Blöcker H., Scholz P., Bagdasarian M., Lanka E. (1986). Molecular cloning of the plasmid RP4 primase region in a multi-host-range *tacP* expression vector. Gene.

