# Supplementary material for: Biosynthesis of the Inner Core of Bordetella pertussis Lipopolysaccharides: Effect of Mutations on LPS Structure, Cell Division, and Toll-like Receptor 4 Activation
Source: Int J Mol Sci. 2023 Dec 9;24(24):17313. doi: 10.3390/ijms242417313 (PMC10743493; doi:10.3390/ijms242417313)
Supplement: Supplementary file 1 [file ijms-24-17313-s001.zip › ijms-2746818-supplementary.pdf]

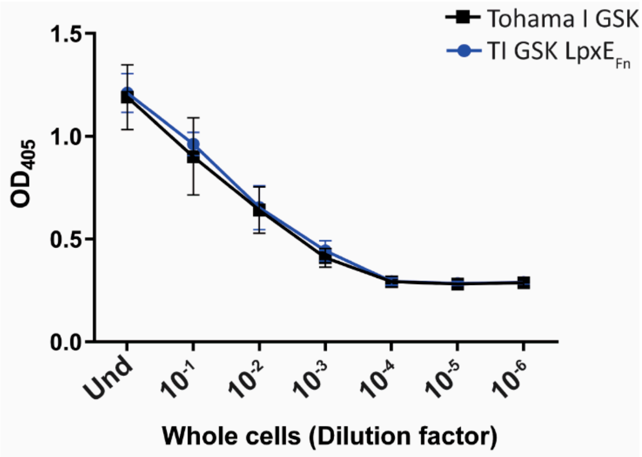

Figure S1. TLR4 activation by whole-cell preparations of strain Tohama I GSK and its LpxE$_{Fn}$-producing derivative. HEK-Blue cells expressing human TLR4 were incubated for 17 h with 10-fold serial dilutions of heat-inactivated whole cells. The OD_{600} of the undiluted suspensions (Und) was 1. Graph shows means and standard deviations of SEAP activity measured at OD_{405} from the supernatants of a single experiment performed in duplicate.

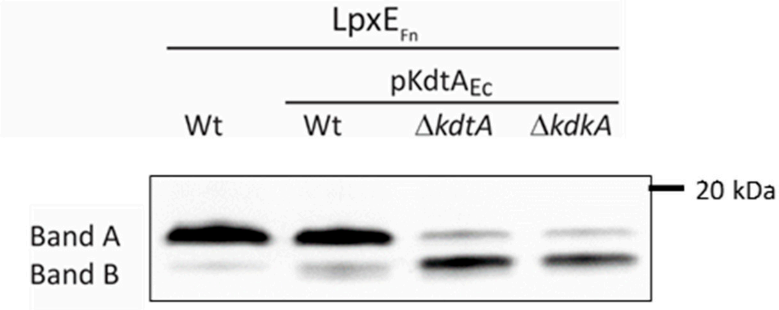

Figure S2. Comparison of LPS from *B. pertussis* strain TI GSK LpxE$_{Fn}$ and its pKdtA$_{Ec}$-containing derivatives**.** OM preparations were analyzed by SDS-PAGE, and LPS was visualized by silver staining. Wt refers to the parental strain Tohama I GSK. The positions of band A and band B are indicated at the left and that of a molecular-weight marker protein at the right.

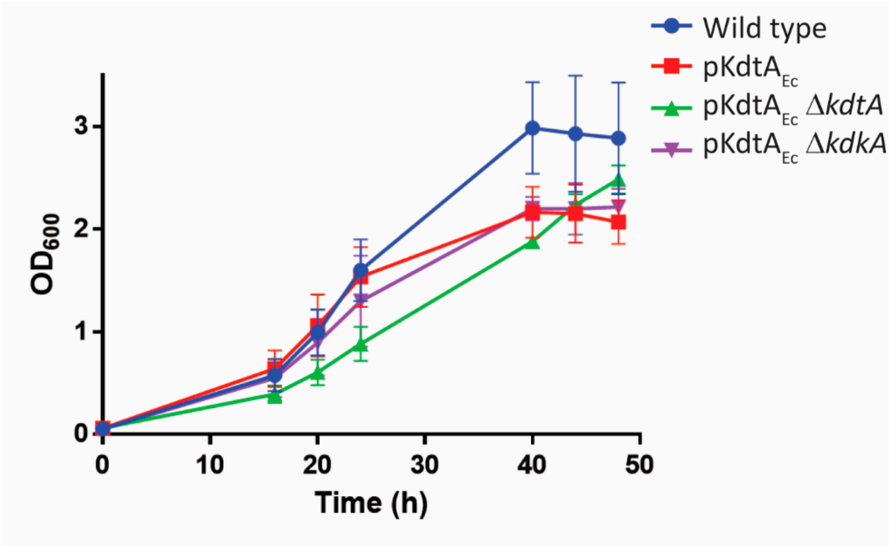

Figure S3. Growth of *B. pertussis* strain B213 (Wild type) and its pKdtA$_{Ec}$-containing derivatives. Optical densities of the cultures were measured at OD_{600} for 48 h. Graph shows values of a single experiment performed in duplicate**.**

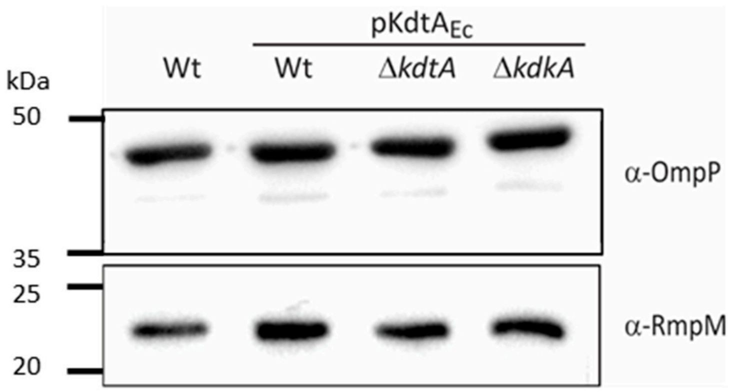

Figure S4. Western blot analysis of relevant OM antigens in strain B213 (Wt) and its pKdtA$_{Ec}$-containing derivatives. Isolated OMs were analyzed by SDS-PAGE and Western blotting with antisera directed against the major porin OmpP and OM-associated protein RmpM. The positions of molecular-weight marker proteins are indicated at the left.

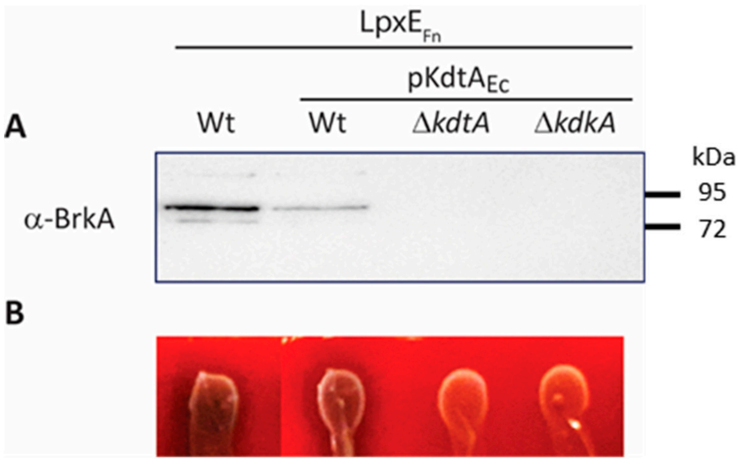

Figure S5. Comparison of Bvg-dependent properties of *B. pertussis* strain TI GSK LpxE$_{Fn}$ and its pKdtA$_{Ec}$-containing derivatives. (**A**) Isolated Oms were analyzed by SDS-PAGE and Western blot analysis with antibodies directed against the autotransporter BrkA. The positions of molecular-weight marker proteins are indicated at the right. (**B**) Hemolytic capacity of the strains. Drops from bacterial cultures were plated on BG-blood agar and incubated for 48 h. The dark halo indicates hemolytic activity. Wt refers to the parental strain Tohama I GSK.

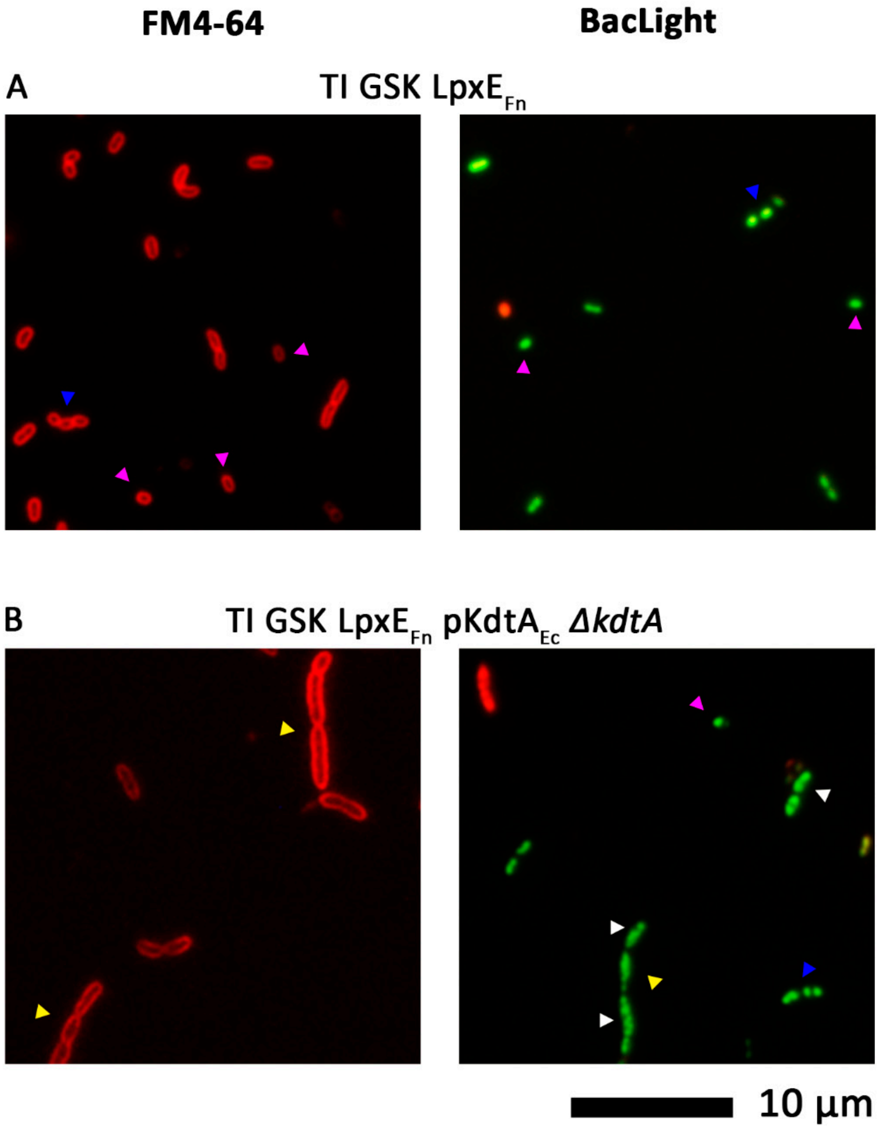

Figure S6. Effect of KdtA$_{Ec}$ production on the morphology of LpxE$_{Fn}$-producing *B. pertussis* cells visualized by fluorescence microscopy. Bacterial cultures of strain TI GSK LpxE$_{Fn}$ (**A**) and its pKdtA$_{Ec}$ Δ*kdtA* derivative (**B**) were stained with either the fluorescent dye FM4-64 (left panels) or Dead/Live BacLight kit (right panels). Scale bar represents 10 µm. Examples of cells showing shorter and more rounded shape than the wild-type cell (purple), short chains (blue), elongated cell chains (yellow), or uneven dye distribution (white) are indicated with colored arrowheads.

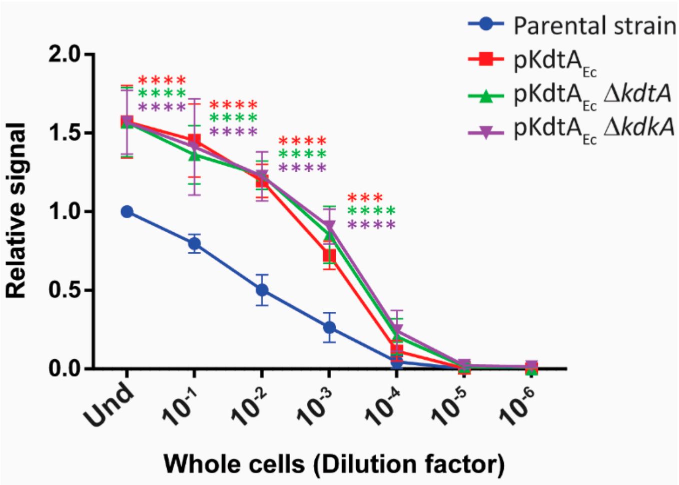

Figure S7. TLR4 activation by whole-cell preparations of strain TI GSK LpxE$_{Fn}$ (Parental strain) and its pKdtA$_{Ec}$-containing derivatives. TLR4-expressing HEK-Blue cells were incubated for 17 h with 10-fold serial dilutions of heat-inactivated whole cells. The OD_{600} of the undiluted suspensions (Und) was 0.15. Graphs show means and standard deviations of relative SEAP activity calculated as the ratio between the signal measured for each dilution with each strain and the signal measured for the undiluted parental strain. Three independent experiments were performed in duplicate. Dilutions of the mutants with activities statistically different from those of the parental strain are indicated with asterisks (***, $p \leq 0.001$; ****, $p \leq 0.0001$).

Table S1 | Bacterial strains and plasmids

Strain / plasmid	Description [a]	Reference
B. pertussis		
B213	Str^R derivative of strain Tohama I	[62]
B213 pKdtA$_{Ec}$	B213 carrying pKdtA$_{Ec}$, Str^R, Amp^R	This study
B213 pKdtA$_{Ec}$ Δ*kdtA*	Δ*kdtA::gem* mutant of B213 pKdtA$_{Ec}$, Str^R, Amp^R, Gem^R	This study
B213 pKdtA$_{Ec}$ Δ*kdkA*	Δ*kdkA::gem* mutant of B213 pKdtA$_{Ec}$, Str^R, Amp^R, Gem^R	This study
B213 Δ*BP2327*	Δ*BP2327::gem* mutant of B213, Str^R, Gem^R	This study
B213 Δ*BP3136*	Δ*BP3136*(*eptB*)*::gem* mutant of B213, Str^R, Gem^R	This study
Tohama I GSK	Str^R derivative of strain Tohama I	GSK[b]
TI GSK LpxF$_{Fn}$	Tohama I GSK carrying pLpxF$_{Fn}$, Str^R, Amp^R	This study
TI GSK LpxE$_{Fn}$	Tohama I GSK carrying *lpxE$_{Fn}$* replacing *lgmB*, Str^R	This study
TI GSK LpxE$_{Fn}$ pKdtA$_{Ec}$	TI GSK LpxE$_{Fn}$ carrying pKdtA$_{Ec}$, Str^R, Amp^R	This study
TI GSK LpxE$_{Fn}$ pKdtA$_{Ec}$ Δ*kdtA*	Δ*kdtA::gem* mutant of TI GSK LpxE$_{Fn}$ pKdtA$_{Ec}$, Str^R, Amp^R, Gem^R	This study
TI GSK LpxE$_{Fn}$ pKdtA$_{Ec}$ Δ*kdkA*	Δ*kdkA::gem* mutant of TI GSK LpxE$_{Fn}$ pKdtA$_{Ec}$, Str^R, Amp^R, Gem^R	This study
E. coli		
DH5α	F$^-$, Δ(*lacZYA-argF)U169 thi-1 hsdR17 gyrA96 recA1 endA1 supE44 relA1 phoA Φ80 dlacZΔM15*	[63]
SM10*λpir*	*thi thr leu fhuA lacY supE recA*::RP4-2-Tc::Mu *λpir* R6K, Kan^R	[64]
W3110	K-12 derivative, F$^-$, λ$^-$, INV *(rrnD$^-$ rrnE)1*	ATCC 27325
Plasmids		
pUC57-Kan Δ*kdtA*	pUC57-Kan derivative harboring *kdtA* knockout construct, Kan^R, Gem^R	This study

pUC57-Kan Δ*kdkA*	pUC57-Kan derivative harboring *kdkA* knockout construct, Kan^R, Gem^R	This study
pUC57-Kan Δ*BP2327*	pUC57-Kan derivative harboring *BP2327* knockout construct, Kan^R, Gem^R	This study
pUC57-Kan Δ*BP3136*	pUC57-Kan derivative harboring *BP3136* knockout construct, Kan^R, Gem^R	This study
pYRC	pBBR1MCS-5 cloning vector containing *lacI*, Gem^R	[65]
pKAS32	Allelic exchange suicide vector, *rpsL*, Amp^R	[66]
pKAS-Δ*kdtA*	pKAS32 derivative harboring *kdtA* knockout construct, Amp^R, Gem^R	This study
pKAS-Δ*kdkA*	pKAS32 derivative harboring *kdkA* knockout construct, Amp^R, Gem^R	This study
pKAS-Δ*BP2327*	pKAS32 derivative harboring *BP2327* knockout construct, Amp^R, Gem^R	This study
pKAS-Δ*BP3136*	pKAS32 derivative harboring *BP3136* knockout construct, Amp^R, Gem^R	This study
pSORTPI	Gem^R derivative of pRTPI, *colE1*, *oriT*, *rpsL*, Amp^R, Gem^R	[67]
pSORTPI-Δ*lgmB*-$LpxE_{Fn}$	pSORTPI harboring *F. novicida lpxE* under *ompP* promoter; construct for insertion as *lgmB* knockout, Amp^R, Gem^R	This study
pMMB67EH	Broad-host-range expression vector, *tac* promoter, Amp^R	[68]
$pKdtA_{Ec}$	pMMB67EH carrying *E. coli kdtA* with a 3’ sequence encoding a His-Tag, Amp^R	This study
$pLpxF_{Fn}$	pMMB67EH carrying *F. novicida lpxF*, Amp^R	This study

[a] Str, streptomycin; Gem, gentamicin; Amp, ampicillin; Kan, kanamycin
[b] GlaxoSmithKline laboratory stocks

Table S2 | PCR primers used in this study

Primer	Sequence (5′→3′) [a]	Description
Fw-XbaI-KdtA-Ec	GCGCGCTCTAGATGCTCGAATTGCTTTACACCGCCC	Primers for amplification of *kdtA E. coli*, introducing 5′-XbaI and 3′-HindIII restriction sites
Rv-KdtA-His-RBS-NdeI-Hind	GCGCGCAAGCTTCATATGTATATCTCCTTCTTAAAGTT**TCA***GTGGTGGTGGTGGTGGTG*ATGCGTTTTCGGTGGCAGGTAAGGTTCCAG	
Fw-Eco81I-GemR	GCGCGCCCTGAGGGACGCACACCGTGGAAA	Primers for amplification of *gem*^{R}, introducing Eco81I restriction sites at both flanks
Rv-GemR-Eco81I	GCGCGCCCTCAGGGCGGCGTTGTGACAATTT	
Fw-XbaI-LpxE	TAATCCTCTAGAATGCTGAAGCAGACCCTCCA	Primers for amplification of codon-optimized *lpxE F. novicida*, introducing 5′-XbaI and 3′-ApaI restriction sites
Rv-LpxE-ApaI	TAAGCTGGGCCC**TTA**GATGATCTCGCGATTGCGCA	

[a] Restriction sites are underlined; stop codon (in reverse primer) is in bold; sequence coding for a His-Tag is in italics.
